# Clinical MetaData ontology: a simple classification scheme for data elements of clinical data based on semantics

**DOI:** 10.1186/s12911-019-0877-x

**Published:** 2019-08-20

**Authors:** Hye Hyeon Kim, Yu Rang Park, Kye Hwa Lee, Young Soo Song, Ju Han Kim

**Affiliations:** 1 Seoul National University Biomedical Informatics (SNUBI), Seoul National University College of Medicine, Seoul, 03080 Republic of Korea; 2 Seoul National University Hospital Biomedical Research Institute, Seoul National University Hospital, Seoul, 03080 Republic of Korea; 3 Department of Biomedical Systems Informatics, Yonsei University College of Medicine, Seoul, 03722 Republic of Korea; 4 Precision Medicine Center, Seoul National University Hospital, Seoul, 03080 Republic of Korea; 5 Department of Pathology, Hanyang University College of Medicine, Seoul, 04763 Republic of Korea; 6 Division of Biomedical Informatics, Seoul National University College of Medicine, 103 Daehak-ro Jongno-gu, Seoul, 03080 Republic of Korea

**Keywords:** Ontology, Common data element, ISO/IEC 11179, Classification

## Abstract

**Background:**

The increasing use of common data elements (CDEs) in numerous research projects and clinical applications has made it imperative to create an effective classification scheme for the efficient management of these data elements. We applied high-level integrative modeling of entire clinical documents from real-world practice to create the Clinical MetaData Ontology (CMDO) for the appropriate classification and integration of CDEs that are in practical use in current clinical documents.

**Methods:**

CMDO was developed using the General Formal Ontology method with a manual iterative process comprising five steps: (1) defining the scope of CMDO by conceptualizing its first-level terms based on an analysis of clinical-practice procedures, (2) identifying CMDO concepts for representing clinical data of general CDEs by examining how and what clinical data are generated with flows of clinical care practices, (3) assigning hierarchical relationships for CMDO concepts, (4) developing CMDO properties (e.g., synonyms, preferred terms, and definitions) for each CMDO concept, and (5) evaluating the utility of CMDO.

**Results:**

We created CMDO comprising 189 concepts under the 4 first-level classes of *Description*, *Event*, *Finding*, and *Procedure*. CMDO has 256 definitions that cover the 189 CMDO concepts, with 459 synonyms for 139 (74.0%) of the concepts. All of the CDEs extracted from 6 HL7 templates, 25 clinical documents of 5 teaching hospitals, and 1 personal health record specification were successfully annotated by 41 (21.9%), 89 (47.6%), and 13 (7.0%) of the CMDO concepts, respectively. We created a CMDO Browser to facilitate navigation of the CMDO concept hierarchy and a CMDO-enabled CDE Browser for displaying the relationships between CMDO concepts and the CDEs extracted from the clinical documents that are used in current practice.

**Conclusions:**

CMDO is an ontology and classification scheme for CDEs used in clinical documents. Given the increasing use of CDEs in many studies and real-world clinical documentation, CMDO will be a useful tool for integrating numerous CDEs from different research projects and clinical documents. The CMDO Browser and CMDO-enabled CDE Browser make it easy to search, share, and reuse CDEs, and also effectively integrate and manage CDEs from different studies and clinical documents.

**Electronic supplementary material:**

The online version of this article (10.1186/s12911-019-0877-x) contains supplementary material, which is available to authorized users.

## Background

Clinical data should be collected in a consistent manner by applying a standardized format so as to facilitate unified data collection, sharing, and integration. Clinical data from multiple sites need to be effectively integrated and compared in order to improve patient care and clinical research. There have been numerous efforts to standardize clinical data. One approach is to construct a common data model, which we call a top-down approach since a top-level knowledge model agreement is applied to the underlying data models of the interoperating parties to ensure successful data exchange [1]. The HL7 Reference Information Model (RIM) and EN 13606 standards are representative data models in the healthcare domain, which include generic reference models of concepts and relationships (e.g., CEN/ISO 13606, openEHR Reference Model, and HL7 RIM) and more-detailed models (e.g., openEHR Archetypes/Templates and HL7 Detailed Clinical Model) [2, 3]. To achieve semantic interoperability, these models utilize connected terms from various standard terminologies such as Unified Medical Language System (UMLS), SNOMED-CT, and LOINC. However, a major problem of this top-down ontology-construction approach is that it takes an unacceptably long time for users to adopt and use them under all possible situations that are encountered in healthcare [4].

Another approach is to simply encourage clinical users to employ standard vocabularies to encode complex real-world concepts. For instance, the patient may say ‘*During the accident I sustained an injury to the back of my head and neck*.’ No concept in UMLS corresponds to the problem ‘*injury to the back of the head and neck*.’ Instead, the problem concept *Injury* (C3263722), the direction *Back* (C0205095), and anatomical locations *Head* (C0018670) and *Neck* (C0027530) must be postcoordinated into an on-the-fly concept [5]. We call this a bottom-up approach because complex real-world concepts are constructed from elementary vocabularies. However, a complex clinical concept is often too sophisticated to be comprehensively encoded in a uniform manner, resulting in postcoordination ambiguities with many different encodings that in turn prohibit semantic interoperability.

The ISO/IEC 11179 international standard for a metadata description and registry (MDR) specifies a metadata model for representing the common data elements (CDEs) that are a logical data unit that provides for data definitions (including an identifier), response values to indicate the value type, and detailed information to represent data concepts and their semantics [6, 7]. The CDE consisted of two parts: a data element concept (DEC) for the meaning of data, and the value domain (VD). For instance, the DEC for a person’s sex is established using the object concepts of *Person* (C0027361) and *Sex* (C1522384), while the VD for sex covers the permissible values of ‘male’ and ‘female.’ The complete CDE is defined by combining the DEC for a person’s sex with VD < male|female> [8].

Well-defined CDEs can be collected and reused as a content standard. Here we propose ‘middle-out’ approach that contrasts with the top-down ontology-construction and bottom-up vocabulary-encoding approaches for achieving semantic interoperability at the level of clinical data elements contained in clinical documents. We first create and use CDEs based on the ISO/IEC 11179 standard for an MDR and then incrementally improve their quality. We call this a ‘middle-out’ approach because CDEs are pragmatically defined at a highly practical level for immediate real-world use, and then we systematically link them ‘up’ to the ontology classifications and ‘down’ to the standard controlled vocabularies. CDEs are designed at a pragmatic or ‘middle’ level and then ‘out’ to the higher ontologies and the lower vocabularies in a systematic way.

There are multiple benefits in using CDEs based on the ISO/IEC 11179 standard, including (1) effective and rapid data collection that reduces the burden on investigators and thereby facilitates their participation in clinical research, (2) improved data sharing and data aggregation due to employing common forms and standard definitions, and (3) higher data quality by providing unified data and their descriptions [9, 10]. Numerous large-scale clinical studies have developed standardized CDEs based on ISO/IEC 11179, such as that from the National Cancer Institute [11, 12], the National Institute of Neurological Disorders and Stroke CDE project [13–15], and other clinical projects with various aims [16, 17]. The National Institutes of Health encourage the use of CDEs [17], which has led to them being deployed in case-report forms (CRFs) and clinical documents [6, 7, 10], and subsequently demonstrating the high effectiveness and usability of employing CDEs. We also implemented ISO/IEC 11179 based on a metadata registry called the Clinico-Histopathological Metadata Registry (CHMR), which contains more than 20,000 highly curated CDEs [18, 19].

One important characteristic of a successful MDR is being able to efficiently search for an appropriate CDE stored in it. For this purpose ISO/IEC 11179 provides a classification scheme (CS) structure for the conceptual classification and identification of data elements. Thus, when constructing an MDR or registering designed CDEs into an MDR, it is also necessary to select or design the contents of the CS using controlled vocabularies [20, 21]. However, most MDRs do not fully utilize or register a CS, and some MDRs support only two or three concept items in each CS for classifying their own metadata. Moreover, most of the CDE browsers that are developed in projects do not apply formal CSs, relying instead on simple keyword-based search engines. Keyword searching suffers from imprecision and ambiguity; for example, documents containing synonyms of the query keywords will not be retrieved, and homonyms cannot be properly managed. An ontology-based search approach can be considered as one example of a semantically enhanced information retrieval method.

We successfully established the CHMR in 2006, since when we have been using it in various clinical trials and research studies [22–24]. The main limitation we have experienced during the long-term use of an MDR is the lack of an ontology that can be used to semantically group, search, and integrate metadata. The objective of the present study was to develop an ontology—called the Clinical Metadata Ontology (CMDO)—for managing, retrieving, classifying, and integrating CDEs with the rich metadata attributes provided by the ISO/IEC 11179 standard. To construct an ontology for use with clinical documents, we used metadata in clinical documents obtained from the real-world clinical setting of a tertiary hospital in South Korea. We evaluated the utility of the developed CMDO using 1 personal health record (PHR) specification [CCR Plus (CCR+)] [22, 24], 6 HL7 templates [25], and 25 common clinical documents from 5 teaching hospitals in South Korea [26].

## Methods

CMDO was developed using one of the appropriate methodologies for conceptual modeling, the General Formal Ontology (GFO) method [27], which is a manual iterative process comprising five steps: (1) defining the scope of CMDO by conceptualizing its first-level terms (or classes), (2) identifying CMDO concepts, (3) assigning hierarchical relationships among CMDO concepts, (4) developing CMDO properties (e.g., synonyms, preferred terms, and definitions) for each CMDO concept, and (5) evaluating the utility of CMDO. All metadata used in our work registered in the CHMR (http://chmr2.snubi.org:8083/chmr/).

### Defining the scope for CMDO

A clinical document is a record of a patient’s medical history and care. Every piece of evidence and background data related to the care can also be documented. It is the most-important source of information for clinical decision-making, communicating between healthcare providers, and addressing legal issues.

Clinical data can be captured, stored, accessed, displayed, and transmitted in clinical practices using clinical documents, which can be designed as a complex structure that comprises a multitude of data elements. We analyzed clinical documents to identify the key concepts that represented the DECs, which became the classes of our ontology. The detailed identifying process is described in the next section.

The typical process of clinical practice can be summarized as follows: The patient is registered at the time of initial contact, with information about his/her health-related problem (history) gathered while also focusing on the current illness, symptoms, and chief complaint. Healthcare providers then perform diagnostic or therapeutic procedures based on the information provided by the patient. This process involving procedures, observations, and testing is repeated until the end of treatment. Events such as admission, discharge, or adverse drug reactions can occur during this interaction process, and the characteristics of these events usually vary between the different general environments of healthcare. By analyzing this series of clinical processes we found that clinical information could be categorized using the following four main terms: (1) *Procedure*, (2) *Finding*, (3) *Event*, and (4) *Description*. These were used as the first-level terms of our ontology: *Procedure* includes all treatments or actions taken to prevent or treat disease, or improve health in other ways; *Finding* includes the collected total of physical and psychological measurements of the patient surveyed or acted on by a medical doctor; *Event* includes all things that happen at a given place and time in a medical situation; and *Description* includes a detailed account of the particular characteristics or symptoms of a patient.

### Identifying CMDO concepts

The CDE is the atomic unit of data and is associated with a DEC (an abstract unit of knowledge for representing semantics) and a VD (representation of data including the data type and permissible values) according to the ISO/IEC 11179 standard.

We identified CMDO concepts using a representative concept (DEC) of data elements (CDEs) from the metadata registry (CHMR). In particular, we selected clinical documents from Seoul National University Hospital (SNUH) related to CDEs from among all of the CDEs in the CHMR in order to query and examine DECs. The frequency of clinical document usage was determined, and only SNUH clinical documents that had been used more than 10 times between January and August 2010 in each hospital department were selected so that the results would be applicable to as many medical disciplines as possible. This approach resulted in 27,109 CDEs being extracted from 663 SNUH clinical documents.

We manually extracted common concepts that were counted more than twice from the DECs while considering whether it was reasonable to subordinate them to first-level terms of CMDO, and chose them as CMDO concepts, which are the child terms of each first-level term. These concepts were reviewed and selected by two medical doctors and two medical informatics researchers. These individuals had an average of 5 years of experience working in family medicine, laboratory medicine, and psychiatry, and were guided to select reasonable subordinate concepts under the four first-level terms of CMDO. For example, we classified *Description* into the following 10 child terms that are readily accepted by most clinicians in SNUH as representing this class: *Advance Directives*, *Alerts*, *Assessment*, *Chief Complaint*, *Demographics*, *Encounter*, *Immunization*, *Past Medical History*, *Present Illness*, and *Vital Signs*. We performed this process of identifying child terms repeatedly until optimal semantic granularity was achieved.

### Assigning relationships among CMDO concepts

**Fig. 1:**
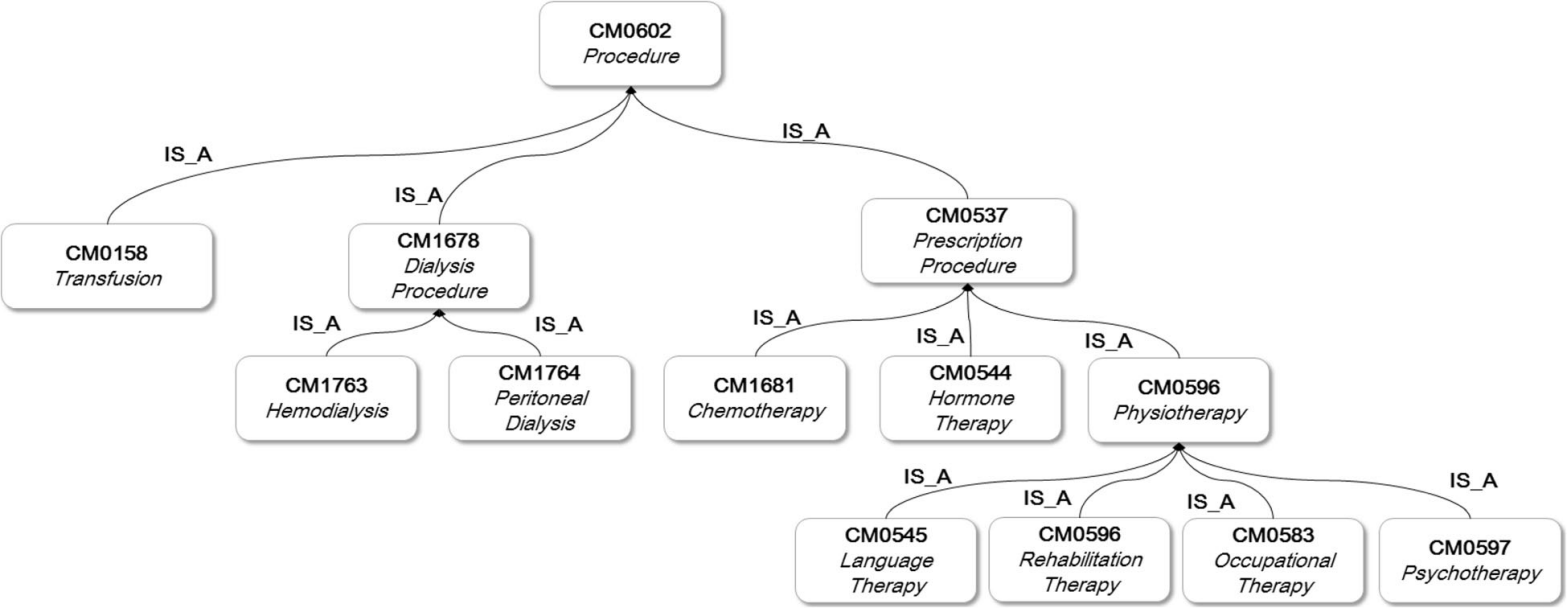
Part of CMDO, formalized as tree structure

CMDO is formally structured as a hierarchical tree structure, with a root value and subtrees of child nodes with a parent node. We assigned an is-a relationship between CMDO concepts by applying the following process: Terms that appeared to be in a subordinates–superiors relationship were determined to be in an is-a relationship involving two medical doctors and two medical informatics researchers. Figure 1 presents a graphical representation of the CMDO classification showing the *Allergy Test* from *Finding* as a parent concept being assigned to *Allergy History* derived largely from *Description*.

### Development of CMDO properties

We created two CMDO properties (synonyms and definitions) for each CMDO concept by referencing the UMLS Metathesaurus and Wikipedia. UMLS has Concept Unique Identifier (CUI), and terms with the same CUI can be grouped together since they are semantically equivalent [28].

When using a UMLS CUI we found synonyms that were flagged in the relationship (REL = ‘same-as’ or ‘possibly-equivalent-to’) column of the MRREL table and in the Term Type in the Source (TTY = ‘SY’) column of the MRCONSO table. We also found definitions that were flagged in the definition (DEF) column of the MRDEF table. For CMDO concepts that were not assigned to a UMLS CUI, we either used Wikipedia or manually described synonyms and definitions used by expert medical doctors.

We also created synonyms for each CMDO concept by reflecting hierarchical structure. During the process of developing hierarchical relationships, identified CMDO concepts were modified to have synonyms to reflect superordinate terms. For example, *Result of Physical Examination* has *Breast* as a child term. In this hierarchical structure, *Breast* refers to a result from a physical examination of the breast, and not to the anatomical structure of the breast. We therefore added the CMDO synonym term as *Breast* to *Result of Physical Examination of Breast*.

### Evaluation scheme

We used two clinical document sets to evaluate CMDO: (1) 6 documents from HL7 templates [Operation Note (2009), Consultation Note (2008), Discharge Summary (2009), History and Physical (2008), Procedure Note (2010), and Progress Note (2010)] and (2) 25 clinical documents, of which 5 were Admission Note, Outpatient Note, Discharge Note, Emergency Note, and Operation Note documents from 5 teaching hospitals in South Korea (SNUH, Pusan National University Hospital, Ajou University Hospital, Chonnam National University Hospital, and Gachon University Gil Hospital). These 5 documents from SNUH and 663 clinical documents mentioned in the Methods section were mutually exclusive. Additional file 1: Table S1 lists the names of the clinical documents that were used for constructing and evaluating CMDO.

To evaluate the suitability of CMDO for facilitating the classification and integration of CDEs, we first applied CMDO annotations to the 96 and 559 CDEs extracted from the 2 clinical document sets. The CMDO annotation process was performed by two independent nurses while considering the most-granular terms in CMDO (where this was possible). Each CDE could be annotated with multiple CMDO concepts. The two nurses who performed the evaluation were certified medical record administrators who had an average of 5 years of work experience. We allowed all cases of agreement or disagreement among these two annotators as following examples. For example, the two annotators chose similar results for the data element ‘Secondary Sexual Character of Adolescent Type Category’ in an Admission Note at Ajou University Hospital, with one nurse choosing *Description|Past history|Developmental history* and the other choosing *Description|Past history|Developmental history* and *Description|Past history|Social history*. However, there was also a case of disagreement, in that for the data element ‘Estimated Blood Loss’ in an Operation Note from the HL7 template, one nurse chose *Procedure|Surgery* and the other chose *Finding|Surgery|Problem*.

Two administrators of medical records separately validated the two CMDO annotation sets. To complete the CMDO annotation process, at least two medical informatics researchers confirmed the above-four CMDO annotation sets and rated their coverage of CMDO into the following categories: adequate, too broad (i.e., first-level terms or general second-level terms), or too specific (i.e., terminal-node terms that were used infrequently). We also examined whether one kind of clinical document (the PHR) could be classified by CMDO.

## Results

### CMDO concepts

The root term of CMDO is *Clinical metadata*, which has four first-level classes. The total number of CMDO concepts is 189. Table 1 lists the statistics of CMDO concepts for each level under the first-level classes. *Finding* is the first-level class with the largest number of child terms (*n* = 82). Additional file 1: Table S2 lists all of the CMDO concepts in their hierarchical structure.

**Table 1:** Statistics of CMDO

First-level class	CMDO level	Number of child terms for each level in each class	Number (%) of child terms for each first-level class
*Description*	1	1	60 (31.9)
	2	10	
	3	9	
	4	38	
	5	1	
	6	1	
*Event*	1	1	15 (8.0)
	2	11	
	3	3	
*Finding*	1	1	82 (43.6)
	2	16	
	3	40	
	4	22	
	5	3	
*Procedure*	1	1	31 (16.5)
	2	14	
	3	12	
	4	4	
Total			188

CMDO provides 459 synonyms for 139 (74.0%) CMDO concepts, and 256 definitions for 188 (100%) CMDO concepts. Most (*n* = 164, 87.7%) of the CMDO concepts were matched to UMLS preferred terms. Most of the UMLS-unmatched CMDO concepts were postcoordinated CMDO concepts or concepts that were too specific, such as *Medication for Skin* and *Gallium Scan*.

### CMDO web service

**Fig. 2:**
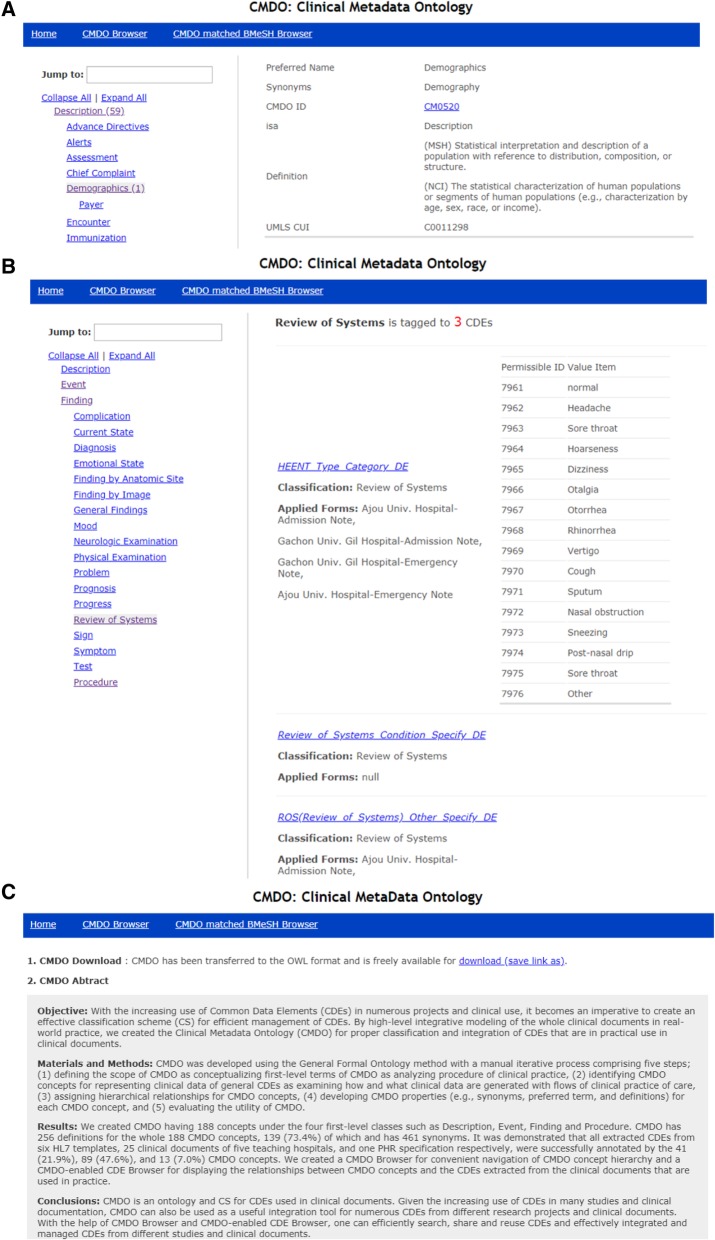
CMDO web services: (**a**) CMDO Browser and (**b**) CMDO-enabled CDE Browser., (**c**) CMDO Download Function (OWL)

To facilitate access to CMDO, we developed the CMDO Browser that provides the CMDO ID, preferred terms, and related properties such as synonyms, definitions, the parent term, and the UMLS CUI (Fig. 2a). The left panel of the CMDO Browser displays lists of CMDO preferred terms arranged as a hierarchical tree that can be navigated by clicking the name of each term to explore CMDO concepts, with their detailed properties displayed in the right panel. The numbers of child terms are indicated in parentheses next to the CMDO concepts. Search functions for CMDO concepts can be made by entering terms in the ‘Jump to:’ box, which also has an autocomplete function.

The mandatory items for CMDO are the preferred terms and CMDO ID. Other properties are optional, which are provided by the CMDO Browser when they do not have null values. We provide CMDO ID with a URL hyperlink to the corresponding CDEs in the CHMR, of which CMDO is one of the CSs.

In the CMDO definition, the abbreviation with three words in brackets indicates the source of the definition in terms of controlled vocabularies in UMLS; for example, *AOT*, *CHV*, *CSP*, *FMA*, *HL7*, *MSH*, and *NCI* represent *Authorized Osteopathic Thesaurus*, *Consumer Health Vocabulary*, *CRISP Thesaurus*, *Foundational Model of Anatomy Ontology*, *HL7 Vocabulary Version 3.0*, *Medical Subject Headings*, and *NCI Thesaurus*, respectively. CMDO definitions that were not obtained from UMLS either did not include brackets or their source information was represented using an indicator such as *Wiki* or *WebMD* [29].

For the purpose of demonstrating the utility of CMDO as a CS for CDEs in various clinical documents, we developed a CMDO-enabled CDE Browser for navigating our 739 CDEs for 31 clinical documents in the CHMR (Fig. 2b). The left panel of the CDE Browser displays shortened names of CMDO terms arranged as a hierarchical tree that can be navigated to explore CDEs tagged with these CMDO terms and related clinical documents by clicking the names. The right panel displays the number of the CDEs tagged with the searched CMDO terms along with the list of CDEs containing the CDE name, CDE definition, and list of clinical documents containing the CDEs. For CDEs having permissible values, it also displays VD information in a tabular format with permissible IDs and items.

CMDO has been implemented in OWL 2 using WebProtégé (version 4.0.0) [30], since this automatically checks the semantic consistency of the data to be entered, which can further improve its scalability. CMDO is freely available for download (Fig. 2c). We also released it in a downloadable form on well-known repository, BioPortal [31, 32].

### Evaluation results

All of the CDEs extracted from two clinical document sets (six HL7 templates and five documents from the five teaching hospitals) were manually annotated with CMDO concepts. Table 2 indicates that 81.3, 96.0, and 100% of the CMDO concepts were rated as being adequately annotated for the 6 HL7 templates, 25 clinical documents, and the CCR+ specification, respectively. However, it was found that the current CMDO could not cover too-detailed CDEs such as ‘*Procedure estimated blood loss specify*’ (and hence were rated as being too broad), which comprised 14.6, 2.7, and 0% of concepts for the 6 HL7 templates, 25 clinical documents, and the CCR+ specification, respectively. It also found that some CMDO concepts were too specific for utilization as a general classification and used for only specific CDEs such as ‘*Consciousness State of Patient Specify*’ (and hence were rated as being too specific), which comprised 4.1, 1.3, and 0% of concepts for the 6 HL7 templates, 25 clinical documents, and the CCR+ specification, respectively. Additional file 1: Table S3 lists the CDEs that were rated as being either too broad or too specific.

**Table 2:** CDEs annotated with CMDO concepts in two clinical document sets

Annotation	HL7 templates (*n* = 6)	Clinical documents from five teaching hospitals (*n* = 25)	CCR+ (*n* = 1)	Total
Adequate	78 (81.3%)	537 (96.0%)	128 (100.0%)	743 (94.9%)
Too broad	14 (14.6%)	15 (2.7%)	0 (0.0%)	29 (3.7%)
Too specific	4 (4.1%)	7 (1.3%)	0 (0.0%)	11 (1.4%)
Total	96 (12.4%)	559 (71.1%)	128 (16.5%)	783 (100.0%)

Data are *n* (%) values

Table 3 lists the distributions of CMDO annotations for the two document sets according to CMDO levels. The 96, 559, and 128 CDEs extracted from the 6 HL7 templates, 25 clinical documents, and the CCR+ specification were annotated to 41 (21.9%), 89 (47.6%), and 13 (7.0%) CMDO concepts, respectively.

**Table 3:** Mapping result of how CMDO concepts in each level in each class are matched to CDEs in different clinical document sets

First-level Class	CMDO level (No. of CMDO concepts)	HL7 templates (*n* = 6)				Clinical documents (*n* = 25)				CCR+ (*n* = 1)			
		No. of matched CMDO concepts		No. of CDEs matched to CMDO concepts		No. of matched CMDO concepts		No. of CDEs matched to CMDO concepts		No. of matched CMDO concepts		No. of CDEs matched to CMDO concepts	
		No.^a^	(%)^b^	Primary ^c^	Multi. ^d^	No.	(%)	Primary	Multi.	No.	(%)	Primary	Multi.
Description	1 (1)	1	100.0	3	3	1	100.0	2	2	0	0.0	0	0
	2 (10)	9	90.0	18	24	6	60.0	48	49	4	40.0	34	34
	3 (9)	4	44.4	13	13	8	88.9	39	41	4	44.4	47	47
	4 (38)	1	2.6	0	5	11	28.5	39	40	0	0.0	0	0
	5 (1)	0	0.0	0	0	0	0.0	0	0	0	0.0	0	0
	5 (1)	0	0.0	0	0	1	100.0	2	2	0	0.0	0	0
All (60)		15	25.0	34	45	27	45.0	130	134	8	13.3	81	81
Event	1 (1)	0	0.0	0	0	0	0.0	0	0	0	0.0	0	0
	2 (11)	4	36.4	5	16	7	63.6	44	67	0	0.0	0	0
	3 (3)	0	0.0	0	0	1	33.3	2	3	0	0.0	0	0
All (15)		4	20.0	5	16	8	53.3	46	70	0	0.0	0	0
Finding	1 (1)	1	100.0	1	1	1	100.0	2	2	0	0.0	0	0
	2 (16)	5	31.3	12	12	10	62.5	106	124	2	12.5	21	21
	3 (40)	7	17.5	10	10	23	57.5	191	202	2	5.0	18	18
	4 (22)	0	0.0	0	0	5	22.7	16	16	0	0.0	0	0
	5 (3)	1	33.3	1	1	1	33.3	1	1	0	0.0	0	0
All (82)		14	17.1	24	24	40	48.8	316	345	4	4.9	39	39
Procedure	1 (1)	1	100.0	11	11	1	100.0	14	14	1	100.0	8	8
	2 (14)	6	42.9	20	21	9	64.2	34	43	0	0.0	0	0
	3 (12)	1	8.3	2	2	4	33.3	12	14	0	0.0	0	0
	4 (4)	0	0.0	0	0	0	0.0	0	0	0	0.0	0	0
All (31)		8	13.8	33	34	14	66.7	60	71	1	3.2	8	8
Total 188		41	21.8	96	119	89	47.3	552	620	13	6.9	128	128

^a^Number of CMDO concepts in each level in each first-level class used to match with DEs

^b^Mapping rate of CMDO concepts per each level in each first-level class

^c^Mapping the CDE with the representative single CMDO concept among multiple annotations

^d^Mapping the CDE with multiple CMDO concepts allowed duplicated counts

The most frequently used CMDO concepts for CMDO-matched CDEs in the 6 HL7 templates and 25 clinical documents were from the *Description* (*n* = 34) and *Finding* (*n* = 316) classes, respectively. Most of the CDEs in both clinical document sets were annotated second-level CMDO concepts of each class, except for the *Description* and *Finding* classes in the 25 clinical documents. Since multiple annotations were allowed, 23 (= 119–96) and 68 (= 620–552) CDEs were annotated by multiple CMDO concepts in both clinical document sets. However, we found no multiple annotations in the CCR+ specification, and the CMDO concepts in the *Description* class (i.e., 8 concepts for 81 CDEs) were frequently used for annotating CDEs from there. Additional file 1: Table S4 lists CMDO concept annotated CDEs from two types of clinical document sets and the CCR+ PHR model.

## Discussion

We have created CMDO as a CS for CDEs created by increasingly popular CDE-related projects with an emphasis on their application to clinical documentation. The most-popular headings extracted from the clinical documents were assigned to CMDO concepts. A relationship between subordinates and superiors among CMDO concepts was defined manually by clinical informatics experts, while the remaining ontology development process was conducted according to the GFO method.

Despite the ongoing and rapid advances in informatics technology, it is still impossible to fully automate the management of the full semantics of clinical documents and their data elements, which is due to the data elements and their values being semantically too diverse and unscalable. In other words, human resources are still needed to manage the full semantics of clinical documents. CMDO may serve as a suitable CS for facilitating interactions between human resources and machines. We can expect CMDO to be useful as (1) a CS for CDEs for clinical documents and CRFs, and (2) a tool for integrating CDEs from diverse clinical documents and CRFs.

The HL7/LOINC Document Ontology (DO) was developed to provide a standard representation of the attributes of clinical documents using a multiaxis structure and to standardize the names of clinical documents as an essential first step toward the optimal use, management, and exchange of documents both within and between institutions [33, 34]. A CS has been provided for clinical documents, and document names have been standardized. However, clinical documents within the same DO classification may be semantically diverse, and those that are in different DO classifications may have a multitude of CDEs in common. Moreover, the structures of real-world clinical documents are constantly evolving. For example, an admission note and discharge summary may share many CDEs such as the chief complaint, present illness, and past history, making them structurally similar but with very different administrative roles. It is therefore necessary to not only classify clinical documents themselves but also classify the CDEs contained within them in order to comprehensively understand and manage such documents. Clinical documents can be automatically classified and automatically managed based on the similarities between the sets of CDEs that they contain. We reviewed the existing upper level ontologies in an attempt to identify an appropriate one for classifying CDEs in clinical documents. However, this was unsuccessful since the available ontologies were either too complicated and had a high complexity (e.g., UMLS) or were not appropriate (e.g., DO); we therefore constructed CMDO.

CMDO provides precise and comprehensive semantic annotations in terms of UMLS, in that each CMDO concept has properties including synonyms and definitions that are mapped to the UMLS CUI, adopting UMLS preferred terms considering CMDO as a part of UMLS in the clinical domain to classify CDEs. Each CMDO concept has properties to show how it can cover the same or similar meanings for discovering semantic correspondences.

We found that CMDO concepts in the *Finding* class exhibited the highest CMDO mapping rate for CDEs in clinical documents. It seems that the CDEs in the 25 real-world clinical documents contained more observational concepts than the CDEs for the exemplar items in the 6 HL7 templates. We also found that the 25 clinical documents contained more-specific CDEs than the 6 HL7 templates, since 3 times as many CMDO terminal concepts were used for mapping the CDEs in the clinical documents.

It should be noted that while CMDO appears to be a feasible ontology for annotating CDEs in clinical documents, but it has the limitation of an insufficient concept coverage due to its contents and granularity; our evaluation revealed that 5% of the CMDO items are either too broad or too specific. Our construction, implementation and use of the ontology also revealed two further limitations. First, when constructing CMDO we did not consider other methods and compare them with the GFO method, instead only considering the GFO since we considered it an appropriate method for conceptualizing ontologies. Second, when using CMDO we adopted an approach that we called ‘middle-out,’ meaning that we systematically linked CDEs up to the ontology and down to the vocabularies. It was implicitly assumed that there were associated appropriate CDEs with the correct CMDO concepts; if this assumption was not valid, it would take more time to match CMDO concepts to CDEs.

In future work we plan to extend the current version of CMDO to also cover the parts that were identified in the evaluation as being either too broad or too specific. We will also apply other ontology methods to CMDO to check it for semantic consistency, and consider adding more concept relationships, such as part-of relationships. We also plan to determine how to simplify the process of using CMDO in order to save time and ensure high usability.

## Conclusion

The sharing, understanding, and integration of data from multiple different domains can be facilitated by standardization. An MDR-based CDE is considered a type of standardized data with specified concept and VDs. This study has demonstrated that a clinical-content-based ontology can be used to identify standardized CDEs. The rapid expansion of CDEs from many types of clinical documents in numerous studies makes CMDO a useful CS and integration tool.

## Additional file

Additional file 1: **Table S1.** List of clinical documents used in building and evaluating CMDO. **Table S2.** List of CMDO concepts and its hierarchical structure. **Table S3.** Full names of definition sources. In form type, HL7 and CDA are represented 6 HL7 templates and 25 clinical documents from 5 teaching hospitals, respectively. **Table S4**. List of CDEs matched to CMDO concepts from two source data. In form type, HL7 and CDA are represented 6 HL7 templates and 25 clinical documents from 5 teaching hospitals, respectively. (DOCX 114 kb)

## Data Availability

The concepts of CMDO developed during the current study are available http://www.snubi.org/software/CMDO/, and http://bioportal.bioontology.org/ontologies/CMDO/.

## **Abbreviations**

CCR +: CCR plus
CDE: Common data elements
CHMR: Clinico-histopathological metadata registry
CMDO: Clinical metadata ontology
CS: Classification scheme
CUI: Concept unique identifier
DEC: Data element concepts
DEF: Definition
DO: Document ontology
MDR: Metadata description and registry
PHR: Personal health record
REL: Relationship label
SNUH: Seoul national university hospital
TTY: Term type in source
UMLS: Unified medical language system
