# Supplementary material for: Clinical MetaData ontology: a simple classification scheme for data elements of clinical data based on semantics
Source: BMC Med Inform Decis Mak. 2019 Aug 20;19:166. doi: 10.1186/s12911-019-0877-x (PMC6701018; doi:10.1186/s12911-019-0877-x)
Supplement: Supplementary file 1 — Table S1. List of clinical documents used in building and evaluating CMDO. Table S2. List of CMDO concepts and its hierarchical structure. Table S3. Full names of definition sources. In form type, HL7 and CDA are represented 6 HL7 templates and 25 clinical documents from 5 teaching hospitals, respectively. Table S4. List of CDEs matched to CMDO concepts from two source data. In form type, HL7 and CDA are represented 6 HL7 templates and 25 clinical documents from 5 teaching hospitals, respectively. (DOCX 114 kb) [file 12911_2019_877_MOESM1_ESM.docx]

**Supplementary Table S1.** List of clinical documents used in building and evaluating CMDO.

* B: Building CMDO, E: Evaluating CMDO

| # | Purpose | Type | (Department) Name of Clinical Document | |
| --- | --- | --- | --- | --- |
| 1 | B | SNUH 663 | Allergy and clinical Immunology | Simple lung function test |
| 2 | B |  | Allergy and clinical Immunology | Other test Report |
| 3 | B |  | Allergy and clinical Immunology | Other Cell stain |
| 4 | B |  | Allergy and clinical Immunology | MBPT |
| 5 | B |  | Allergy and clinical Immunology | Lysine aspirin challenge test |
| 6 | B |  | Allergy and clinical Immunology | intranasal endoscopy_RESULT |
| 7 | B |  | Allergy and clinical Immunology | Inhalation antigen skin test Report |
| 8 | B |  | Allergy and clinical Immunology | Induced Sputum Examination |
| 9 | B |  | Allergy and clinical Immunology | Food skin reaction test |
| 10 | B |  | Allergy and clinical Immunology | Exercise-induced test |
| 11 | B |  | Allergy and clinical Immunology | Eosinophil stain |
| 12 | B |  | Allergy and clinical Immunology | Drug skin reaction test |
| 13 | B |  | Allergy and clinical Immunology | Drug Oral challenge test |
| 14 | B |  | Allergy and clinical Immunology | Cerecoxib oral-induced test |
| 15 | B |  | Allergy and clinical Immunology | Capsaicin-induced test Report |
| 16 | B |  | Allergy and clinical Immunology | Aspirin-induced oral test |
| 17 | B |  | Allergy and clinical Immunology | Allergy and clinical Immunology_progress note_New_Free Text |
| 18 | B |  | Allergy and clinical Immunology | Allergy and clinical Immunology_progress note_FREE TEXT |
| 19 | B |  | Allergy and clinical Immunology | Allergy and clinical Immunology _progress note_Asthma_rhinitis_chronic cough |
| 20 | B |  | Allergy and clinical Immunology | Allergy and clinical Immunology _Outpatient_Asthma_rhinitis_chronic cough |
| 21 | B |  | Allergy and clinical Immunology | Allergy and clinical Immunology _Discharge_discharge |
| 22 | B |  | Anesthesiology | Surgery |
| 23 | B |  | Anesthesiology | Fibromyalgia diagnosis criteria |
| 24 | B |  | Anesthesiology | Diagnostic criteria for complex regional pain syndrome |
| 25 | B |  | Anesthesiology | Anesthesiology_progress note_Pain center |
| 26 | B |  | Anesthesiology | Anesthesiology_progress note_FREE TEXT |
| 27 | B |  | Anesthesiology | Anesthesiology_progress |
| 28 | B |  | Anesthesiology | Anesthesiology_PI_pain |
| 29 | B |  | Anesthesiology | Anesthesiology_PI_leg pain |
| 30 | B |  | Anesthesiology | Anesthesiology_PI_headache |
| 31 | B |  | Anesthesiology | Anesthesiology_Outpatient_Pain center |
| 32 | B |  | Anesthesiology | Anesthesiology_Inpatient/Outpatient/Emergency_FREE TEXT |
| 33 | B |  | Anesthesiology | Anesthesiology_first medical examination_exam_general |
| 34 | B | SNUH 663 | Anesthesiology | Anesthesiology_discharge |
| 35 | B |  | Anesthesiology(anesthesia) | Anesthesiology(anesthesia)_progress note_New_Free Text |
| 36 | B |  | Anesthesiology(anesthesia) | Anesthesiology(anesthesia)_PI_FREE TEXT |
| 37 | B |  | Anesthesiology(anesthesia) | Anesthesiology(anesthesia)_Inpatient/Outpatient/Emergency_FREE TEXT |
| 38 | B |  | ‎Breast clinic | Upfront ZO-FAST 462 |
| 39 | B |  | ‎Breast clinic | ‎Breast clinic_progress note_New_Free Text |
| 40 | B |  | ‎Breast clinic | ‎Breast clinic_progress note_FREE TEXT |
| 41 | B |  | ‎Breast clinic | ‎Breast clinic_progress note_Breast biopsy |
| 42 | B |  | ‎Breast clinic | ‎Breast clinic_Outpatient/Emergency_FREE TEXT |
| 43 | B |  | ‎Breast clinic | ‎Breast clinic_Outpatient/Emergency_Default |
| 44 | B |  | Cardiothoracic Surgery | Valve registry |
| 45 | B |  | Cardiothoracic Surgery | Non-Small Cell Lung Cancer Sheet |
| 46 | B |  | Cardiothoracic Surgery | Lung cancer_surgery_preoperative |
| 47 | B |  | Cardiothoracic Surgery | Lung cancer_surgery_postoperative |
| 48 | B |  | Cardiothoracic Surgery | Lung cancer_surgery_intraoperative |
| 49 | B |  | Cardiothoracic Surgery | Lung cancer_report |
| 50 | B |  | Cardiothoracic Surgery | Intern_Inpatient_PI |
| 51 | B |  | Cardiothoracic Surgery | Follow Up Card(Esophagous Cancer) |
| 52 | B |  | Cardiothoracic Surgery | Follow Up Card (Lung Cancer) |
| 53 | B |  | Cardiothoracic Surgery | first medical examination_PI_FREETEXT |
| 54 | B |  | Cardiothoracic Surgery | Extracorporeal circulation (adult, child) |
| 55 | B |  | Cardiothoracic Surgery | Cardiothoracic Surgery-Discharge summary-freetext |
| 56 | B |  | Cardiothoracic Surgery | Cardiothoracic Surgery_symptom |
| 57 | B |  | Cardiothoracic Surgery | Cardiothoracic Surgery_progress note_FREE TEXT |
| 58 | B |  | Cardiothoracic Surgery | Cardiothoracic Surgery_Outpatient/Emergency_New_Free Text |
| 59 | B |  | Cardiothoracic Surgery | Cardiothoracic Surgery_Outpatient/Emergency_FREE TEXT |
| 60 | B |  | Cardiothoracic Surgery | Cardiothoracic Surgery_operative note_FREE TEXT |
| 61 | B |  | Cardiothoracic Surgery | Cardiothoracic Surgery_Inpatient/Outpatient/Emergency_FREE TEXT |
| 62 | B |  | Cardiothoracic Surgery | Cardiothoracic Surgery_Discharge_general |
| 63 | B |  | Cardiothoracic Surgery | Cardiothoracic Surgery_Discharge_FREE TEXT |
| 64 | B |  | Cardiothoracic Surgery | Cardiothoracic Surgery_Discharge_Discharge summary |
| 65 | B |  | Cardiothoracic Surgery | Cardiothoracic Surgery_01. s/p CABG |
| 66 | B |  | Circulation medicine | Head-up Tilt Test Report |
| 67 | B |  | Circulation medicine | Exercise_ECG_Result_Report |
| 68 | B |  | Circulation medicine | Electrophysiological test_radiofrequency catheter ablation |
| 69 | B |  | Circulation medicine | Echocardiography (Cardiothoracic) Report |
| 70 | B |  | Circulation medicine | ECG_Result_Report |
| 71 | B |  | Circulation medicine | Circulatory short term obligation / Discharge summary |
| 72 | B |  | Circulation medicine | Circulation medicine_progress note_post-surgery echocardiogram test Report |
| 73 | B |  | Circulation medicine | Circulation medicine_progress note_plain |
| 74 | B |  | Circulation medicine | Circulation medicine_progress note_Default |
| 75 | B |  | Circulation medicine | Circulation medicine_PI_chest pain |
| 76 | B | SNUH 663 | Circulation medicine | Circulation medicine_PI_CAG |
| 77 | B |  | Circulation medicine | Circulation medicine_Outpatient_Default_상세 |
| 78 | B |  | Circulation medicine | Circulation medicine_Inpatient/Outpatient/Emergency_exam_brief2 |
| 79 | B |  | Circulation medicine | Circulation medicine_Discharge_formula |
| 80 | B |  | Circulation medicine | Circulation medicine_consult_plain |
| 81 | B |  | Circulation medicine | Artificial pacemaker implantation |
| 82 | B |  | Circulation medicine | AMI Report |
| 83 | B |  | Circulation medicine | 25hr BP monitoring Report |
| 84 | B |  | Circulation medicine | 24hr ECG_Result_Report |
| 85 | B |  | Circulation medicine | 24hr BP monitoring Report |
| 86 | B |  | Circulation medicine | [Cath & CAG] 1 Summary |
| 87 | B |  | Dermatology | Psoriasis treatment of psoriasis (psoriasis) |
| 88 | B |  | Dermatology | Patch Test [Ⅰ] |
| 89 | B |  | Dermatology | Insolation Report |
| 90 | B |  | Dermatology | Dermatology_surgery |
| 91 | B |  | Dermatology | Dermatology_progress note_FREE TEXT |
| 92 | B |  | Dermatology | Dermatology_progress note_ |
| 93 | B |  | Dermatology | Dermatology_PI_whole body skin lesion |
| 94 | B |  | Dermatology | Dermatology_PI_Urticaria |
| 95 | B |  | Dermatology | Dermatology_PI_skin lesion |
| 96 | B |  | Dermatology | Dermatology_PI_scalp skin lesions |
| 97 | B |  | Dermatology | Dermatology_PI_pruritus |
| 98 | B |  | Dermatology | Dermatology_PI_herpes zoster |
| 99 | B |  | Dermatology | Dermatology_PI_hair loss |
| 100 | B |  | Dermatology | Dermatology_PI_form DEFAULT |
| 101 | B |  | Dermatology | Dermatology_PI_facial skin lesion |
| 102 | B |  | Dermatology | Dermatology_PI_acne |
| 103 | B |  | Dermatology | Dermatology_Outpatientprogress |
| 104 | B |  | Dermatology | Dermatology_Outpatientfirst medical examination_ |
| 105 | B |  | Dermatology | Dermatology_Outpatient_ |
| 106 | B |  | Dermatology | Dermatology_first medical examination_PE |
| 107 | B |  | Dermatology | Dermatology_discharge |
| 108 | B |  | Division of infectious diseases | Infectious diseases_PI_VDRL |
| 109 | B |  | Division of infectious diseases | Infectious diseases_Full |
| 110 | B |  | Division of infectious diseases | Infectious diseases_Consultation_default |
| 111 | B |  | Division of infectious diseases | Division of infectious diseases_progress note_progress note |
| 112 | B |  | Emergency medicine | Emergency transfer patient report |
| 113 | B |  | Emergency medicine | Emergency medicine_progress |
| 114 | B |  | Emergency medicine | Emergency medicine_PI_stomachache |
| 115 | B |  | Emergency medicine | Emergency medicine_PI_plain |
| 116 | B |  | Emergency medicine | Emergency medicine_PI_fever |
| 117 | B |  | Emergency medicine | Emergency medicine_PI_DOA |
| 118 | B | SNUH 663 | Emergency medicine | Emergency medicine_discharge |
| 119 | B |  | Emergency medicine | Emergency medicine exam set (ver.0307.5) |
| 120 | B |  | Emergency medicine(EMO) | Pre-hospital breathing difficulty assessment tool |
| 121 | B |  | Emergency medicine(EMO) | In-depth investigation of cerebrovascular disease (stroke) |
| 122 | B |  | Emergency medicine(EMO) | In-Depth Injury Surveillance-Brain/Face/Spine |
| 123 | B |  | Emergency medicine(EMO) | Hospital Remittance Report |
| 124 | B |  | Emergency medicine(EMO) | Emergency transfer patient report |
| 125 | B |  | Emergency medicine(EMO) | Emergency Severity Index |
| 126 | B |  | Emergency medicine(EMO) | Emergency room-based impairment monitoring system |
| 127 | B |  | Emergency medicine(EMO) | Emergency medicine(EMO)_progress note_New_Free Text |
| 128 | B |  | Emergency medicine(EMO) | Emergency medicine(EMO)_progress note_Emergency progress |
| 129 | B |  | Emergency medicine(EMO) | Emergency medicine(EMO)_PI_FREE TEXT |
| 130 | B |  | Emergency medicine(EMO) | Emergency medicine(EMO)_Outpatient/Emergency_New_Free Text |
| 131 | B |  | Emergency medicine(EMO) | Emergency medicine(EMO)_Outpatient/Emergency_FREE TEXT |
| 132 | B |  | Emergency medicine(EMO) | Emergency medicine(EMO)_Outpatient/Emergency_Emergency center_first medical examination |
| 133 | B |  | Emergency medicine(EMO) | Emergency medicine(EMO)_Inpatient/Outpatient/Emergency_FREE TEXT |
| 134 | B |  | Emergency medicine(EMO) | Emergency Airway Registry_200803 |
| 135 | B |  | Emergency medicine(EMO) | Deep examination of cardiovascular disease (acute myocardial infarction) |
| 136 | B |  | Emergency medicine(EMO) | CPR registry (out-of-hospital) |
| 137 | B |  | Emergency medicine(EMO) | CPR registry (in-hospital) |
| 138 | B |  | Emergency medicine(EMO) | Chest Pain Work-Up sheet |
| 139 | B |  | Emergency medicine(EMO) | Central Nerve System Injury Disability Surveillance |
| 140 | B |  | Emergency(AER) | Emergency(AER)_progress note_New_Free Text |
| 141 | B |  | Emergency(AER) | Emergency(AER)_PI_FREE TEXT |
| 142 | B |  | Emergency(AER) | Emergency(AER)_Outpatient/Emergency_New_Free Text |
| 143 | B |  | Emergency(AER) | Emergency(AER)_Outpatient/Emergency_FREE TEXT |
| 144 | B |  | Emergency(AER) | Emergency(AER)_Inpatient/Outpatient/Emergency_FREE TXET |
| 145 | B |  | Endocrinology | Orthostatic blood pressure test |
| 146 | B |  | Endocrinology | first medical examination_PI_FREETEXT |
| 147 | B |  | Endocrinology | Endocrinology_progress note_New_Free Text |
| 148 | B |  | Endocrinology | Endocrinology_progress note_FREE TEXT |
| 149 | B |  | Endocrinology | Endocrinology_progress note_ENDO_progress_default |
| 150 | B |  | Endocrinology | Endocrinology_progress note |
| 151 | B |  | Endocrinology | Endocrinology_Outpatient/Emergency_New_Free Text |
| 152 | B |  | Endocrinology | Endocrinology_Outpatient/Emergency_FREE TEXT |
| 153 | B |  | Endocrinology | Endocrinology_consult |
| 154 | B | SNUH 663 | Endocrinology | Endocrine atherosclerosis test (carotid ultrasound) |
| 155 | B |  | Endocrinology | ENDO_PI_diabetes |
| 156 | B |  | Endocrinology | ENDO_EXAM |
| 157 | B |  | Endocrinology | ENDO_consult |
| 158 | B |  | Endocrinology | Basal metabolism measurement Report |
| 159 | B |  | Family medicine | Nicotine dependence self diagnosis (Fagerstorm) |
| 160 | B |  | Family medicine | International prostate symptom score (I-PSS) |
| 161 | B |  | Family medicine | Early dementia test K-MMSE |
| 162 | B |  | Family medicine | Depression screening test |
| 163 | B |  | Gastroenterology | Upper gastrointestinal endoscopic guide |
| 164 | B |  | Gastroenterology | Ultrasound Endoscopy Report |
| 165 | B |  | Gastroenterology | UBT Report |
| 166 | B |  | Gastroenterology | therapeutic endoscopy_result |
| 167 | B |  | Gastroenterology | therapeutic endoscopy(throat)_result |
| 168 | B |  | Gastroenterology | therapeutic endoscopy(large intestine)_result |
| 169 | B |  | Gastroenterology | Lower gastrointestinal tract (large intestine) Endoscopy Report |
| 170 | B |  | Gastroenterology | Liver biopsy and PEIT |
| 171 | B |  | Gastroenterology | Gastroenterology_short term_inpatient_PI |
| 172 | B |  | Gastroenterology | Gastroenterology_progress note_New_Free Text |
| 173 | B |  | Gastroenterology | Gastroenterology_progress note_Gastroenterology progress note |
| 174 | B |  | Gastroenterology | Gastroenterology_progress note_FREE TEXT |
| 175 | B |  | Gastroenterology | Gastroenterology_Outpatient_PI_dyspepsia |
| 176 | B |  | Gastroenterology | Gastroenterology_Outpatient_PI_acute abdominal pain |
| 177 | B |  | Gastroenterology | Gastroenterology_Outpatient/Emergency_New_Free Text |
| 178 | B |  | Gastroenterology | Gastroenterology_Inpatient/Outpatient/Emergency_FREE TEXT |
| 179 | B |  | Gastroenterology | Gastroenterology_Inpatient/Outpatient/Emergency_exam Outpatient |
| 180 | B |  | Gastroenterology | Gastroenterology_Discharge_Gastroenterology Discharge summary |
| 181 | B |  | Gastroenterology | Gastroenterology_Discharge_FREE TEXT |
| 182 | B |  | Gastroenterology | Gastroenterology_Consultation |
| 183 | B |  | Gastroenterology | Gastroenterology Outpatient PI UGI bleeding |
| 184 | B |  | Gastroenterology | Gastroenterology Outpatient PI liver |
| 185 | B |  | Gastroenterology | Gastroenterology Outpatient PI jaundice |
| 186 | B |  | Gastroenterology | first medical examination_PI_FREETEXT |
| 187 | B |  | Gastroenterology | Esophageal manometry |
| 188 | B |  | Gastroenterology | Endoscopy Test Report |
| 189 | B |  | Gastroenterology | Endoscopic submucosal dissection |
| 190 | B |  | Gastroenterology | Endoscopic retrograde cholangiopancreatography (ERCP) |
| 191 | B |  | Gastroenterology | CLO Report |
| 192 | B |  | Gastroenterology | Biofeedback (Constipation) |
| 193 | B |  | Gastroenterology | Acute intestinal acidity test report |
| 194 | B |  | Gastroenterology | (short term) short term_admission note |
| 195 | B |  | Health Promotion Center | (health examination)Physical test result_old version |
| 196 | B |  | Health Promotion Center | (health examination)Nutrition evaluation |
| 197 | B | SNUH 663 | Health Promotion Center | (health examination)Nutrition consult report |
| 198 | B |  | Health Promotion Center | (health examination)Health Report |
| 199 | B |  | Health Promotion Center | (health examination)Fundus examination Report |
| 200 | B |  | Health Promotion Center | (health examination)Energy Consumption evaluation |
| 201 | B |  | Health Promotion Center | (health examination)ECG test Report |
| 202 | B |  | Hemato-oncology | SNUH Medical Oncology Solid Tumor Form |
| 203 | B |  | Hemato-oncology | SNUH Hematology Flow Sheet |
| 204 | B |  | Hemato-oncology | Pharmacogenomics sampling |
| 205 | B |  | Hemato-oncology | HMO_PE_simple version |
| 206 | B |  | Hemato-oncology | Hemato-oncology_progress note_progress |
| 207 | B |  | Hemato-oncology | Hemato-oncology_progress note |
| 208 | B |  | Hemato-oncology | Hemato-oncology_PI_FREETEXT |
| 209 | B |  | Hemato-oncology | Hemato-oncology_PI_CBC abnormality |
| 210 | B |  | Hemato-oncology | Hemato-oncology_PI_anticancer |
| 211 | B |  | Hemato-oncology | Hemato-oncology_PI_anemia |
| 212 | B |  | Hemato-oncology | Hemato-oncology_Outpatient/Emergency_FREE TEXT |
| 213 | B |  | Hemato-oncology | Hemato-oncology_Outpatient |
| 214 | B |  | Hemato-oncology | Hemato-oncology_Inpatient/Outpatient/Emergency_FREE TEXT |
| 215 | B |  | Hemato-oncology | Hemato-oncology_Discharge_discharge |
| 216 | B |  | Hemato-oncology | Hemato-oncolog_PI_FREE TEXT |
| 217 | B |  | Hemato-oncology | Clinical trial Report |
| 218 | B |  | Hemato-oncology | Clinical test body test and specific symptom |
| 219 | B |  | Hemato-oncology | (short term) tumor patients_short term Report |
| 220 | B |  | Internal medicine | Bronchoscopy Previsit Sheet |
| 221 | B |  | Laboratory medicine | Laboratory medicine_PI_FREE TEXT |
| 222 | B |  | Nephrology | UNCC Follow Up sheet |
| 223 | B |  | Nephrology | Transplantation-recipient (cadaveric) |
| 224 | B |  | Nephrology | Transplantation - recipient (living related) |
| 225 | B |  | Nephrology | Transplantation - Donor sheet |
| 226 | B |  | Nephrology | TPL Face Sheet |
| 227 | B |  | Nephrology | SGA Form |
| 228 | B |  | Nephrology | PKD Face Sheet |
| 229 | B |  | Nephrology | Nephrology_progress_New_Free Text |
| 230 | B |  | Nephrology | Nephrology_progress note_progress |
| 231 | B |  | Nephrology | Nephrology_PI_replacement plan |
| 232 | B |  | Nephrology | Nephrology_PI_renal biopsy |
| 233 | B |  | Nephrology | Nephrology_PI_proteinuria |
| 234 | B |  | Nephrology | Nephrology_PI_edema |
| 235 | B |  | Nephrology | Nephrology_PI_dyspnea |
| 236 | B |  | Nephrology | Nephrology_PI_CRF |
| 237 | B |  | Nephrology | Nephrology_PI_ARF |
| 238 | B |  | Nephrology | Nephrology_Outpatientfirst medical examination/Emergency_New_Free Text |
| 239 | B |  | Nephrology | Nephrology_Inpatient/Outpatient/Emergency_FREE TEXT |
| 240 | B | SNUH 663 | Nephrology | Nephrology_Inpatient/Outpatient/Emergency_exam_A/P_form |
| 241 | B |  | Nephrology | Nephrology_Inpatient/Outpatient/Emergency_exam |
| 242 | B |  | Nephrology | Nephrology_discharge _FREE TEXT |
| 243 | B |  | Nephrology | Nephrology_discharge _formula |
| 244 | B |  | Nephrology | Lupus nephritis_Activity index |
| 245 | B |  | Nephrology | Lupus nephritis _ Evaluation of quality of life survey (SF-36) |
| 246 | B |  | Nephrology | Kidney Emergency Care Clinic (UNCC) |
| 247 | B |  | Nephrology | Hematuria Face Sheet |
| 248 | B |  | Neurology | Wada Report |
| 249 | B |  | Neurology | Transcranial Doppler Report |
| 250 | B |  | Neurology | Stroke Report |
| 251 | B |  | Neurology | SNUH Eye Movement Test Report |
| 252 | B |  | Neurology | progress_PI_free text |
| 253 | B |  | Neurology | PI_evaluation |
| 254 | B |  | Neurology | PI_changedmentalstatus |
| 255 | B |  | Neurology | PHx~A/P general |
| 256 | B |  | Neurology | Neurology_progress note_Stroke I |
| 257 | B |  | Neurology | Neurology_progress note_Parkinson |
| 258 | B |  | Neurology | Neurology_progress note_New_Free Text |
| 259 | B |  | Neurology | Neurology_progress note_FREE TEXT |
| 260 | B |  | Neurology | Neurology_progress note_Epilepsy_Regular follow up |
| 261 | B |  | Neurology | Neurology_Outpatient/Emergency_FREE TEXT |
| 262 | B |  | Neurology | Neurology_Inpatient/Outpatient/Emergency_FREE TEXT |
| 263 | B |  | Neurology | Neurology (NR)_progress note_PI_free text(BP) |
| 264 | B |  | Neurology | Jolly Test |
| 265 | B |  | Neurology | Induced potential test |
| 266 | B |  | Neurology | first medical examination_PI_FREETEXT |
| 267 | B |  | Neurology | electroneuromyography_RESULT |
| 268 | B |  | Neurology | electroneurography_RESULT |
| 269 | B |  | Neurology | EEG Analysis Sheet II |
| 270 | B |  | Neurology | EEG Analysis Sheet I |
| 271 | B |  | Neurology | Discharge |
| 272 | B |  | Neurology | Diagnosis of Cognitive Impairment Test Report |
| 273 | B |  | Neurology | Cognitive domain Specific Test Report |
| 274 | B |  | Neurology | Cerebellar Function Test |
| 275 | B |  | Neurology | Blink reflex |
| 276 | B |  | Neurology | ANS function test |
| 277 | B |  | Neurology | Analysis Sheet Of Video-EEG Monitering |
| 278 | B |  | Neurology | Abnormal motion scale |
| 279 | B |  | Neuropsychiatry | YMRS |
| 280 | B |  | Neuropsychiatry | YBOCS |
| 281 | B |  | Neuropsychiatry | UKU S/E scale |
| 282 | B |  | Neuropsychiatry | SOAP_rev |
| 283 | B | SNUH 663 | Neuropsychiatry | SOAP |
| 284 | B |  | Neuropsychiatry | Psychosocial assessment of organ transplantation |
| 285 | B |  | Neuropsychiatry | Psychosocial assessment of organ donors |
| 286 | B |  | Neuropsychiatry | Psychological assessment Report (psychological diagnosis-doppelt) |
| 287 | B |  | Neuropsychiatry | Psychological assessment Report (psychological diagnosis) |
| 288 | B |  | Neuropsychiatry | Psychological assessment Report (personality assessment) |
| 289 | B |  | Neuropsychiatry | PANSS |
| 290 | B |  | Neuropsychiatry | Neuropsychiatry_progress note_Outpatient_CGI-BP |
| 291 | B |  | Neuropsychiatry | Neuropsychiatry_progress note_New_Free Text |
| 292 | B |  | Neuropsychiatry | Neuropsychiatry_progress note_FREE TEXT |
| 293 | B |  | Neuropsychiatry | Neuropsychiatry_progress note_CLP |
| 294 | B |  | Neuropsychiatry | Neuropsychiatry_progress note_CGI |
| 295 | B |  | Neuropsychiatry | Neuropsychiatry_PI_CLP |
| 296 | B |  | Neuropsychiatry | Neuropsychiatry_Outpatient/Emergency_New_Free Text |
| 297 | B |  | Neuropsychiatry | Neuropsychiatry_Outpatient/Emergency_FREE TEXT |
| 298 | B |  | Neuropsychiatry | Neuropsychiatry_Inpatient/Outpatient/Emergency_FREE TEXT |
| 299 | B |  | Neuropsychiatry | Neuropsychiatry_Inpatient/Outpatient/Emergency_CLP |
| 300 | B |  | Neuropsychiatry | Neuropsychiatry_first medical examination_PI |
| 301 | B |  | Neuropsychiatry | Neuropsychiatry_Discharge |
| 302 | B |  | Neuropsychiatry | Neuropsychiatry (NP)_Outpatient |
| 303 | B |  | Neuropsychiatry | Neurocognitive test report |
| 304 | B |  | Neuropsychiatry | Multifaceted toughness test and other special test |
| 305 | B |  | Neuropsychiatry | Montgomery Asberg Depression Rating Scale (MADRS) Form |
| 306 | B |  | Neuropsychiatry | MMSE-KC |
| 307 | B |  | Neuropsychiatry | MMSE-K |
| 308 | B |  | Neuropsychiatry | K-WAIS |
| 309 | B |  | Neuropsychiatry | Isolation and compulsive test Report |
| 310 | B |  | Neuropsychiatry | Intern_Inpatient_PI |
| 311 | B |  | Neuropsychiatry | first medical examination_PI_FREETEXT |
| 312 | B |  | Neuropsychiatry | Epilepsy Neuropsychological Assessment Report (before and after surgery) |
| 313 | B |  | Neuropsychiatry | Electroconvulsive Therapy(electrotherapy) |
| 314 | B |  | Neuropsychiatry | CDR (dementia rating scale test) |
| 315 | B |  | Neuropsychiatry | Behavior limitation Reason Statement |
| 316 | B |  | Neuropsychiatry | Beck Deprssion Inventory |
| 317 | B |  | Neuropsychiatry | Beck Anxiety Inventory |
| 318 | B |  | Neuropsychiatry | Appraisal assessment Report |
| 319 | B |  | Neuropsychiatry | AIMS |
| 320 | B |  | Neuropsychiatry | ADS(Attention Deficit Hyperactivity Disorder; ADHD Diagnostic System) |
| 321 | B |  | Neurosurgery | VP/KP Record |
| 322 | B |  | Neurosurgery | Spontaneous activities |
| 323 | B |  | Neurosurgery | Spine Clinic Survey (Cervical Pain) Report |
| 324 | B |  | Neurosurgery | Neurosurgery_surgery_brain_general |
| 325 | B |  | Neurosurgery | Neurosurgery_progress note_Outpatient_New_Brain Common |
| 326 | B | SNUH 663 | Neurosurgery | Neurosurgery_progress note_New_Free Text |
| 327 | B |  | Neurosurgery | Neurosurgery_progress note_FREE TEXT |
| 328 | B |  | Neurosurgery | Neurosurgery_progress |
| 329 | B |  | Neurosurgery | Neurosurgery_Outpatient/Emergency_New_Free Text |
| 330 | B |  | Neurosurgery | Neurosurgery_Outpatient/Emergency_FREE TEXT |
| 331 | B |  | Neurosurgery | Neurosurgery_operative note_FREE TEXT |
| 332 | B |  | Neurosurgery | Neurosurgery_Inpatient_progress_spine_lumbar_exam |
| 333 | B |  | Neurosurgery | Neurosurgery_Inpatient/Outpatient/Emergency_FREE TEXT |
| 334 | B |  | Neurosurgery | Neurosurgery_first medical examination_exam_spine |
| 335 | B |  | Neurosurgery | Neurosurgery_first medical examination_exam_brain |
| 336 | B |  | Neurosurgery | Neurosurgery_Discharge_FREE TEXT |
| 337 | B |  | Neurosurgery | Neurosurgery_discharge _brain_cranial_AVF |
| 338 | B |  | Neurosurgery | Korean Version of ODI |
| 339 | B |  | Neurosurgery | IOM |
| 340 | B |  | Neurosurgery | Intern_Inpatient_PI |
| 341 | B |  | Neurosurgery | first medical examination_PI_FREETEXT |
| 342 | B |  | Neurosurgery | Cervical Artificial Disc Protocol |
| 343 | B |  | Neurosurgery | Cerebral aneurysm embolization |
| 344 | B |  | Neurosurgery | (short term) Cerebral aneurysm embolization |
| 345 | B |  | Nuclear medicine | short term_Inpatient Record |
| 346 | B |  | Nuclear medicine | Nuclear medicine_progress note_New_Free Text |
| 347 | B |  | Nuclear medicine | Nuclear medicine_Outpatient/Emergency_New_Free Text |
| 348 | B |  | Nuclear medicine | Nuclear medicine_discharge |
| 349 | B |  | OB/GYN | UDS REPORT |
| 350 | B |  | OB/GYN | Semen Analysis Report |
| 351 | B |  | OB/GYN | POP [Outpatient] |
| 352 | B |  | OB/GYN | Polycystic Ovarian Patients_Sonographic Finding Report |
| 353 | B |  | OB/GYN | PISQ-12 |
| 354 | B |  | OB/GYN | PFDI-20 |
| 355 | B |  | OB/GYN | ONCOCHART |
| 356 | B |  | OB/GYN | OB/GYN_progress note_progress |
| 357 | B |  | OB/GYN | OB/GYN_progress note_OB/GYN |
| 358 | B |  | OB/GYN | OB/GYN_progress note_New_Free Text |
| 359 | B |  | OB/GYN | OB/GYN_progress note_FREE TEXT |
| 360 | B |  | OB/GYN | OB/GYN_PI_vaginal spotting |
| 361 | B |  | OB/GYN | OB/GYN_PI_vaginal discharge |
| 362 | B |  | OB/GYN | OB/GYN_PI_vaginal bleeding |
| 363 | B |  | OB/GYN | OB/GYN_PI_postpartum |
| 364 | B |  | OB/GYN | OB/GYN_PI_postop bleeding |
| 365 | B |  | OB/GYN | OB/GYN_PI_postcoital bleeding |
| 366 | B |  | OB/GYN | OB/GYN_PI_pain |
| 367 | B |  | OB/GYN | OB/GYN_PI_mass |
| 368 | B |  | OB/GYN | OB/GYN_PI_infertility |
| 369 | B | SNUH 663 | OB/GYN | OB/GYN_PI_fever |
| 370 | B |  | OB/GYN | OB/GYN_PI_dysuria |
| 371 | B |  | OB/GYN | OB/GYN_PI_dysmenorrhea |
| 372 | B |  | OB/GYN | OB/GYN_PI_cyst |
| 373 | B |  | OB/GYN | OB/GYN_PI_chemotherapy |
| 374 | B |  | OB/GYN | OB/GYN_PI_bearing down sense |
| 375 | B |  | OB/GYN | OB/GYN_PI_ascites |
| 376 | B |  | OB/GYN | OB/GYN_PI_anorexia |
| 377 | B |  | OB/GYN | OB/GYN_PI_amenorrhea |
| 378 | B |  | OB/GYN | OB/GYN_PI_abdominal distension |
| 379 | B |  | OB/GYN | OB/GYN_operative note_pelviscopy |
| 380 | B |  | OB/GYN | OB/GYN_operative note_general |
| 381 | B |  | OB/GYN | OB/GYN_operative note_FREE TEXT |
| 382 | B |  | OB/GYN | OB/GYN_operative note_childbirth |
| 383 | B |  | OB/GYN | OB/GYN_Inpatient/Outpatient/Emergency_OB/GYN |
| 384 | B |  | OB/GYN | OB/GYN_Inpatient/Outpatient/Emergency_FREE TEXT |
| 385 | B |  | OB/GYN | OB/GYN_Discharge_general |
| 386 | B |  | OB/GYN | OAB_SUI [Outpatient progress note] |
| 387 | B |  | OB/GYN | KHQ |
| 388 | B |  | OB/GYN | IUI protocol |
| 389 | B |  | OB/GYN | Intern_Inpatient_PI |
| 390 | B |  | OB/GYN | Infertility Chart |
| 391 | B |  | OB/GYN | In vitro baby program for Outpatient and short term admission note |
| 392 | B |  | OB/GYN | Image Report |
| 393 | B |  | OB/GYN | Hirsutism scoring record |
| 394 | B |  | OB/GYN | FISH Report |
| 395 | B |  | OB/GYN | first medical examination_PI_FREETEXT |
| 396 | B |  | OB/GYN | Fetal US Screening protocol(20-24weeks) |
| 397 | B |  | OB/GYN | Endometriosis Chart |
| 398 | B |  | OB/GYN | Cytogenetic Report |
| 399 | B |  | OB/GYN | American Fertility Society Classification Of Endometriosis : 1985 |
| 400 | B |  | OB/GYN | 3D Hysterosalpingosonography Protocol |
| 401 | B |  | OB/GYN | (short term) GYN Oncology short term record |
| 402 | B |  | Ophthalmology | surgery-Retina |
| 403 | B |  | Ophthalmology | Outpatientprogress-common |
| 404 | B |  | Ophthalmology | Outpatient_general new |
| 405 | B |  | Ophthalmology | Outpatient_common |
| 406 | B |  | Ophthalmology | Ophthalmology_progress note_Outpatientprogress_ Glaucoma |
| 407 | B |  | Ophthalmology | Ophthalmology_progress note_New_Free Text |
| 408 | B |  | Ophthalmology | Ophthalmology_progress note_common progress note |
| 409 | B |  | Ophthalmology | Ophthalmology_progress note |
| 410 | B |  | Ophthalmology | Ophthalmology_PI_Glaucoma |
| 411 | B |  | Ophthalmology | Ophthalmology_PI_General |
| 412 | B | SNUH 663 | Ophthalmology | Ophthalmology_PI_FREETEXT |
| 413 | B |  | Ophthalmology | Ophthalmology_Outpatient/Emergency_New_Free Text |
| 414 | B |  | Ophthalmology | Ophthalmology_operative note_Retina |
| 415 | B |  | Ophthalmology | Ophthalmology_operative note_LASIK |
| 416 | B |  | Ophthalmology | Ophthalmology_operative note_FREE TEXT |
| 417 | B |  | Ophthalmology | Ophthalmology_operative note_Exotropia |
| 418 | B |  | Ophthalmology | Ophthalmology_operative note_cataract3 |
| 419 | B |  | Ophthalmology | Ophthalmology_Inpatient/Outpatient/Emergency_Glaucoma |
| 420 | B |  | Ophthalmology | Ophthalmology_Inpatient/Outpatient/Emergency_general first medical examination |
| 421 | B |  | Ophthalmology | Ophthalmology_Inpatient/Outpatient/Emergency_General |
| 422 | B |  | Ophthalmology | Ophthalmology_Inpatient/Outpatient/Emergency_FREE TEXT |
| 423 | B |  | Ophthalmology | Ophthalmology_Discharge_General |
| 424 | B |  | Ophthalmology | Ophthalmology Prescription |
| 425 | B |  | Ophthalmology | Low vision test |
| 426 | B |  | Ophthalmology | Keratoconus index |
| 427 | B |  | Ophthalmology | first medical examination_Cataract |
| 428 | B |  | Ophthalmology | first medical examination_ Retina |
| 429 | B |  | Ophthalmology | first medical examination_ Glaucoma |
| 430 | B |  | Ophthalmology | Auto Refracto Keratometry |
| 431 | B |  | Ophthalmology | (short) Strabismus |
| 432 | B |  | Ophthalmology | (short) Lacrimal Surgery |
| 433 | B |  | Ophthalmology | (short) Glaucoma |
| 434 | B |  | Ophthalmology | (short) Eyelid Surgery |
| 435 | B |  | Ophthalmology | (short) Epiblepharon |
| 436 | B |  | Ophthalmology | (short) Enucleation/Evisceration |
| 437 | B |  | Ophthalmology | (short) Cataract |
| 438 | B |  | Orthopedics_Surgery | Shoulder P/E Sheet |
| 439 | B |  | Orthopedics_Surgery | Orthopedics_Surgery_surgery_SIMPLE |
| 440 | B |  | Orthopedics_Surgery | Orthopedics_Surgery_progress note_New_Free Text |
| 441 | B |  | Orthopedics_Surgery | Orthopedics_Surgery_progress note_Hip |
| 442 | B |  | Orthopedics_Surgery | Orthopedics_Surgery_progress note_FREE TEXT |
| 443 | B |  | Orthopedics_Surgery | Orthopedics_Surgery_PN_TUMOR |
| 444 | B |  | Orthopedics_Surgery | Orthopedics_Surgery_Outpatient_Hip |
| 445 | B |  | Orthopedics_Surgery | Orthopedics_Surgery_Outpatient/Emergency_New_Free Text |
| 446 | B |  | Orthopedics_Surgery | Orthopedics_Surgery_Outpatient/Emergency_FREE TEXT |
| 447 | B |  | Orthopedics_Surgery | Orthopedics_Surgery_operative note_FREE TEXT |
| 448 | B |  | Orthopedics_Surgery | Orthopedics_Surgery_Inpatient/Outpatient/Emergency_FREE TEXT |
| 449 | B |  | Orthopedics_Surgery | Orthopedics_Surgery_discharge |
| 450 | B |  | Orthopedics_Surgery | Motion analysis |
| 451 | B |  | Orthopedics_Surgery | Foot_epidemiology test |
| 452 | B |  | Orthopedics_Surgery | first medical examination_PI_FREETEXT |
| 453 | B |  | Orthopedics_Surgery | [KNEE] Knee Chart Outpatient progress |
| 454 | B |  | Orthopedics_Surgery | [KNEE] Knee Chart Outpatient first medical examination |
| 455 | B | SNUH 663 | Orthopedics_Surgery | [KNEE Sports] KT-2000 Arthrometer |
| 456 | B |  | Orthopedics_Surgery | [HIP] WOMAC |
| 457 | B |  | Orthopedics_Surgery | [HAND test] Blood test |
| 458 | B |  | Otolaryngology | Vestibular Maneuver |
| 459 | B |  | Otolaryngology | Tinnitogram |
| 460 | B |  | Otolaryngology | TASTE TEST |
| 461 | B |  | Otolaryngology | Otolaryngology_progress note_New_Free Text |
| 462 | B |  | Otolaryngology | Otolaryngology_operative note_FREE TEXT |
| 463 | B |  | Otolaryngology | Otolaryngology_operative note_Cochlear implantation |
| 464 | B |  | Otolaryngology | Otolaryngology_Inpatient/Outpatient/Emergency_FREE TEXT |
| 465 | B |  | Otolaryngology | Otolaryngology_Discharge_FREE TEXT |
| 466 | B |  | Otolaryngology | Nasalance Data (Nasometer) |
| 467 | B |  | Otolaryngology | Laryngeal Function test-Integrated Report |
| 468 | B |  | Otolaryngology | Language Assessment Report I |
| 469 | B |  | Otolaryngology | KVSS test II (comprehensive) |
| 470 | B |  | Otolaryngology | first medical examination_PI_FREETEXT |
| 471 | B |  | Otolaryngology | ENT_surgery_Tonsillectomy with V-tube |
| 472 | B |  | Otolaryngology | ENT_surgery_general |
| 473 | B |  | Otolaryngology | ENT_surgery_ESS |
| 474 | B |  | Otolaryngology | ENT_PI_Nose |
| 475 | B |  | Otolaryngology | ENT_PI_Head and Neck |
| 476 | B |  | Otolaryngology | ENT_PI_Ear |
| 477 | B |  | Otolaryngology | ENT_Outpatientprogress_Plain |
| 478 | B |  | Otolaryngology | ENT_Intern_Note |
| 479 | B |  | Otolaryngology | ENT_discharge |
| 480 | B |  | Otolaryngology | ENT_consult_blank |
| 481 | B |  | Otolaryngology | Eardrum motion measurement test |
| 482 | B |  | Otolaryngology | Butanol Threshold test |
| 483 | B |  | Otolaryngology | (short term) Tonsillectomy & Adenoidectomy Ventilation Tube Inseration |
| 484 | B |  | Otolaryngology | (short term) Sinus endoscopic surgery |
| 485 | B |  | Otolaryngology | (short term) Septoplasty |
| 486 | B |  | Otolaryngology | (short term) Laryngomicrosurgery |
| 487 | B |  | Plastic surgery | PS-PI |
| 488 | B |  | Plastic surgery | PS-operative note |
| 489 | B |  | Plastic surgery | PS-first medical examination |
| 490 | B |  | Plastic surgery | PS-discharge -surgery |
| 491 | B |  | Plastic surgery | PS_Outpatientprogress |
| 492 | B |  | Plastic surgery | PS_Outpatient |
| 493 | B |  | Plastic surgery | Plastic surgery_progress note_FREE TEXT |
| 494 | B |  | Plastic surgery | Plastic surgery_PI_FREE TEXT |
| 495 | B |  | Plastic surgery | Plastic surgery_operative note_default |
| 496 | B |  | Plastic surgery | Plastic surgery_operative note_(allergy)_Default |
| 497 | B |  | Plastic surgery | Plastic surgery_operative note |
| 498 | B | SNUH 663 | Plastic surgery | Plastic surgery_Inpatient/Outpatient/Emergency_FREE TEXT |
| 499 | B |  | Plastic surgery | Plastic surgery_first medical examination |
| 500 | B |  | Pulmonology | Pulmonology_progress note_progress |
| 501 | B |  | Pulmonology | Pulmonology_progress note_New_Free Text |
| 502 | B |  | Pulmonology | Pulmonology_progress note_FREE TEXT |
| 503 | B |  | Pulmonology | Pulmonology_PI_Intern_Inpatient |
| 504 | B |  | Pulmonology | Pulmonology_PI_first medical examination_FREETEXT |
| 505 | B |  | Pulmonology | Pulmonology_PI_first medical examination_dyspnea |
| 506 | B |  | Pulmonology | Pulmonology_Outpatient/Emergency_New_Free Text |
| 507 | B |  | Pulmonology | Pulmonology_Outpatient/Emergency_Default |
| 508 | B |  | Pulmonology | Pulmonology_Inpatient/Outpatient/Emergency_FREE TEXT |
| 509 | B |  | Pulmonology | Pulmonology_Discharge_FREE TEXT |
| 510 | B |  | Pulmonology | Pulmonology_Discharge_discharge |
| 511 | B |  | Pulmonology | Pulmonary Function Lab Report |
| 512 | B |  | Pulmonology | Pre-bronchoscopy Visit Report |
| 513 | B |  | Pulmonology | Exercise load Pulmonary function test result Report |
| 514 | B |  | Pulmonology | Bronchoscopy Test Report |
| 515 | B |  | Pulmonology | Bronchoscopy Previsit Sheet |
| 516 | B |  | Radiology | Diagnostic radiology_progress note_FREE TEXT |
| 517 | B |  | Radiology | Diagnostic radiology_Outpatient/Emergency_FREE TEXT |
| 518 | B |  | Radiology | contrast medium side effects |
| 519 | B |  | Rehabilitation | (PT)Thermoelectric Therapy Progress Note |
| 520 | B |  | Rehabilitation | (PT)Therapeutic Exercise Progress Note |
| 521 | B |  | Rehabilitation | (PT)ROM Test Report |
| 522 | B |  | Rehabilitation | (PT)Gross motor function measurement(GMFM) Report |
| 523 | B |  | Rehabilitation | (PT)Exercise treatment Assessment Report |
| 524 | B |  | Rehabilitation | (OT)Progress Note |
| 525 | B |  | Rehabilitation | (OT)Pediatric Occupational Therapy Evaluation |
| 526 | B |  | Rehabilitation | (OT)Pediatric Jebsen Hand Function Test |
| 527 | B |  | Rehabilitation | (OT)OCCUPATIONAL THERAPY |
| 528 | B |  | Rehabilitation | (OT)NEW JEBSEN HAND FUNCTION TEST |
| 529 | B |  | Rehabilitation | (OT)Modified Barthel Index Score |
| 530 | B |  | Rehabilitation | (OT)MMT & ROM |
| 531 | B |  | Rehabilitation | (OT)MMSE-K(Mini-Mental State Exam - Korea) |
| 532 | B |  | Rehabilitation | (OT)JHFT(Writing) |
| 533 | B |  | Rehabilitation | (OT)JEBSEN HAND FUNCTION TEST |
| 534 | B |  | Rehabilitation | Thermoelectric Therapy Evaluation Site |
| 535 | B |  | Rehabilitation | Standard Spinal Cord Injury Neurological Level |
| 536 | B |  | Rehabilitation | Pre_diagnosis_Report |
| 537 | B |  | Rehabilitation | O'connor finger dexterity test |
| 538 | B |  | Rehabilitation | Mini Mental State Examination Report |
| 539 | B |  | Rehabilitation | Lymphedema Outpatient sheet |
| 540 | B |  | Rehabilitation | Han's Stroke Mobility Scale |
| 541 | B | SNUH 663 | Rehabilitation | Gait Analysis |
| 542 | B |  | Rehabilitation | Cybex-knee |
| 543 | B |  | Rehabilitation | Berg's Balance Scale |
| 544 | B |  | Rehabilitation | Balance Test |
| 545 | B |  | Rheumatology | Rheumatology_progress note_New_Free Text |
| 546 | B |  | Rheumatology | Rheumatology_progress note_FREE TEXT |
| 547 | B |  | Rheumatology | Rheumatology_progress note_ rheumatism |
| 548 | B |  | Rheumatology | Rheumatology_Outpatient/Emergency_New_Free Text |
| 549 | B |  | Rheumatology | Rheumatology_Outpatient/Emergency_FREE TEXT |
| 550 | B |  | Rheumatology | Polarizing Microscopic Examination of Synovial Fluid |
| 551 | B |  | Rheumatology | Musculoskeletal Ultrasonic Test Report |
| 552 | B |  | Surgery | Surgery_progress note_Stomach |
| 553 | B |  | Surgery | Surgery_progress note_HBP |
| 554 | B |  | Surgery | Surgery_progress note_FREE TEXT |
| 555 | B |  | Surgery | Surgery_progress note_Colorectal malignancy |
| 556 | B |  | Surgery | Surgery_PI_freetext |
| 557 | B |  | Surgery | Surgery_Outpatient_Stomach |
| 558 | B |  | Surgery | Surgery_Outpatient_Colorectal malignancy |
| 559 | B |  | Surgery | Surgery_Outpatient/Emergency_New_Free Text |
| 560 | B |  | Surgery | Surgery_Outpatient/Emergency_HBP |
| 561 | B |  | Surgery | Surgery_Outpatient/Emergency_FREE TEXT |
| 562 | B |  | Surgery | Surgery_operative note_Vascular |
| 563 | B |  | Surgery | Surgery_operative note_Surgery_operative note_local anesthesia |
| 564 | B |  | Surgery | Surgery_operative note_Stomach |
| 565 | B |  | Surgery | Surgery_operative note_renal transplantation |
| 566 | B |  | Surgery | Surgery_operative note_operative note (Stomach) |
| 567 | B |  | Surgery | Surgery_operative note_Neck |
| 568 | B |  | Surgery | Surgery_operative note_FREE TEXT |
| 569 | B |  | Surgery | Surgery_operative note_Default |
| 570 | B |  | Surgery | Surgery_operative note_Breast_recurred |
| 571 | B |  | Surgery | Surgery_operative note_Breast_primary |
| 572 | B |  | Surgery | Surgery_operative note_(Vascular) |
| 573 | B |  | Surgery | Surgery_operative note_(Hernia) |
| 574 | B |  | Surgery | Surgery_operative note_(HBP) |
| 575 | B |  | Surgery | Surgery_operative note_(appendectomy) |
| 576 | B |  | Surgery | Surgery_operative note_(anal operation) |
| 577 | B |  | Surgery | Surgery_operative note_ (Neck and Thyroid) |
| 578 | B |  | Surgery | Surgery_Inpatient/Outpatient/Emergency_FREE TEXT |
| 579 | B |  | Surgery | Surgery_Inpatient/Outpatient/Emergency_(default) |
| 580 | B |  | Surgery | Surgery_Discharge_FREE TEXT |
| 581 | B |  | Surgery | PI (HBP) |
| 582 | B |  | Surgery | PI (breast) |
| 583 | B |  | Surgery | PI (Benign anal diseases) |
| 584 | B | SNUH 663 | Surgery | operative note (Surgery default) |
| 585 | B |  | Surgery | operative note (Colorectal) |
| 586 | B |  | Surgery | Intern_Inpatient_PI |
| 587 | B |  | Surgery | first medical examination_PI_FREETEXT |
| 588 | B |  | Surgery(department) | Liver Donor PostOP Course |
| 589 | B |  | Surgery(department) | Discharge summary(Head & Neck, Thyroid) |
| 590 | B |  | Surgery(department) | Discharge summary (Vascular disease) |
| 591 | B |  | Surgery(department) | Discharge summary (Stomach) |
| 592 | B |  | Surgery(department) | Discharge summary (Liver cancer) |
| 593 | B |  | Surgery(department) | Discharge summary (Laparoscopic Cholecystectomy) |
| 594 | B |  | Surgery(department) | Discharge summary (HBP) |
| 595 | B |  | Surgery(department) | Discharge summary (Colorectal cancer) |
| 596 | B |  | Surgery(department) | Discharge summary (Breast) |
| 597 | B |  | Surgery(department) | Biliary Endoscopy Test Report |
| 598 | B |  | Therapeutic Radiology | Therapeutic Radiology Summery |
| 599 | B |  | Therapeutic Radiology | Therapeutic Radiology diagram |
| 600 | B |  | Therapeutic Radiology | Target volume summary & Prescription(E) |
| 601 | B |  | Therapeutic Radiology | Systemic treatment Plan |
| 602 | B |  | Therapeutic Radiology | Radiation oncology_progress note_Post-Therapeutic Radiology |
| 603 | B |  | Therapeutic Radiology | Radiation oncology_progress note_New_Free Text |
| 604 | B |  | Therapeutic Radiology | Radiation oncology_progress note_middle of treatment_progress_SOAP |
| 605 | B |  | Therapeutic Radiology | Radiation oncology_progress note_Interact-Therapeutic Radiology |
| 606 | B |  | Therapeutic Radiology | Radiation oncology_progress note_FREE TEXT |
| 607 | B |  | Therapeutic Radiology | Radiation oncology_progress note_after treatment_progress_SOAP |
| 608 | B |  | Therapeutic Radiology | Radiation oncology_Outpatient/Emergency_SOAP |
| 609 | B |  | Therapeutic Radiology | Radiation oncology_Outpatient/Emergency_New_Free Text |
| 610 | B |  | Therapeutic Radiology | Radiation oncology_operative note_FREE TEXT |
| 611 | B |  | Therapeutic Radiology | Radiation oncology_Inpatient/Outpatient/Emergency_FREE TEXT |
| 612 | B |  | Therapeutic Radiology | Patient_Medical_History |
| 613 | B |  | Therapeutic Radiology | IMRT QA |
| 614 | B |  | Therapeutic Radiology | first medical examination_PI_FREETEXT |
| 615 | B |  | Therapeutic Radiology | DVH |
| 616 | B |  | Therapeutic Radiology | DRR |
| 617 | B |  | Therapeutic Radiology | Dosimetry Report-Scan |
| 618 | B |  | Therapeutic Radiology | Dosimetry Report-RTP |
| 619 | B |  | Therapeutic Radiology | Dose Profile-Sagittal |
| 620 | B |  | Therapeutic Radiology | Dose Profile-Coronal |
| 621 | B |  | Therapeutic Radiology | Dose Profile-Axial |
| 622 | B |  | Therapeutic Radiology | Beam Direction-3D view |
| 623 | B |  | Urology | Urology_progress note_New_Free Text |
| 624 | B |  | Urology | Urology_PI_VOIDING SYMPTOM |
| 625 | B |  | Urology | Urology_PI_URO PAIN |
| 626 | B |  | Urology | Urology_PI_MASS |
| 627 | B | SNUH 663 | Urology | Urology_PI_INCONTINENCE |
| 628 | B |  | Urology | Urology_PI_IMPOTENCE |
| 629 | B |  | Urology | Urology_PI_HYDRONEPHROSIS |
| 630 | B |  | Urology | Urology_PI_HEMATURIA |
| 631 | B |  | Urology | Urology_PI_DYSURIA |
| 632 | B |  | Urology | Urology_PI_CHEMOTHERAPY |
| 633 | B |  | Urology | Urology_PI_BLADDER TUMOR |
| 634 | B |  | Urology | Urology_PI: varicocele |
| 635 | B |  | Urology | Urology_PI: prostate |
| 636 | B |  | Urology | Urology_PI: infertility |
| 637 | B |  | Urology | Urology_PI: fever |
| 638 | B |  | Urology | Urology_Inpatient/Outpatient/Emergency_FREE TEXT |
| 639 | B |  | Urology | Urology_first medical examination_exam |
| 640 | B |  | Urology | Urology_Discharge_FREE TEXT |
| 641 | B |  | Urology | progress note |
| 642 | B |  | Urology | Other Requested Reply Report |
| 643 | B |  | Urology | first medical examination_PI_FREETEXT |
| 644 | B |  | Urology | Discharge |
| 645 | B |  | Urology | (Urination_Treatment) Thoracotomy Synchronization OAB (First Game) Evaluation |
| 646 | B |  | Urology | (Urination_survey) KHQ (kings) |
| 647 | B |  | Urology | (Urination_survey) I-QOL (I call) |
| 648 | B |  | Urology | (Urination_survey) International prostate symptom score table IPSS (N) |
| 649 | B |  | Urology | (Urination_survey) International Prostate Symptom Score Table (IPSS) |
| 650 | B |  | Urology | (Urination_survey) ICSI & ICPI (Interstitial cystitis O'Leary survey paper) |
| 651 | B |  | Urology | (Urination_diagnosis) Urodynamic Study Report |
| 652 | B |  | Urology | (Urination_diagnosis) SNUH Free Uroflowmetry Report |
| 653 | B |  | Urology | (Urination_diagnosis) Potassium test (interstitial cystitis) |
| 654 | B |  | Urology | (Urination_diagnosis) 1hr Pad Test |
| 655 | B |  | Urology | (Urination_ therapy) CIC education (retraining) |
| 656 | B |  | Urology | (surgery) Urology Endoscopy Test Report |
| 657 | B |  | Urology | (surgery) SWL report |
| 658 | B |  | Urology | (surgery) LUT evaluation summary (ver 1.0: 20080609) |
| 659 | B |  | Urology | (sexual function) Semen Evaluation |
| 660 | B |  | Urology | (sexual function) PET Report |
| 661 | B |  | Urology | (sexual function) Erection function survey |
| 662 | B |  | Urology | (sexual function) AMS Questionnaire |
| 663 | B |  | Urology | (sexual function) ADAM Questionnaire |
| 664 | E | 6 documents from HL7 templates | Operation Note (2009) | |
| 665 | E |  | Consultation Note (2008) | |
| 666 | E |  | Discharge Summary (2009) | |
| 667 | E |  | History and Physical (2008) | |
| 668 | E |  | Procedure Note (2010) | |
| 669 | E |  | Progress Note (2010) | |
| 670 | E | 25 clinical documents, of which were 5 documents 5 teaching hospitals in Korea | Admission Note | |
| 671 | E |  | Outpatient Note | |
| 672 | E |  | Discharge Note | |
| 673 | E |  | Emergency Note | |
| 674 | E |  | Operation Note | |
| 675 | E | 1 PHR | CCR+ | |

**Supplementary Table S2.** List of CMDO concepts and its hierarchical structure

| **#** | **CMDO concept** | **CMDO concepts with hierarchical structure** |
| --- | --- | --- |
| 1 | Description | Description |
| 2 | Advance Directives | Description\|Advance Directives |
| 3 | Alerts | Description\|Alerts |
| 4 | Assessment | Description\|Assessment |
| 5 | Chief Complaint | Description\|Chief Complaint |
| 6 | Demographics | Description\|Demographics |
| 7 | Payer | Description\|Demographics\|Payor |
| 8 | Encounter | Description\|Encounter |
| 9 | Immunization | Description\|Immunization |
| 10 | Past Medical History | Description\|Past Medical History |
| 11 | Developmental History | Description\|Past Medical History\|Developmental History |
| 12 | Disease History | Description\|Past Medical History\|Disease History |
| 13 | Allergy History | Description\|Past Medical History\|Disease History\|Allergy History |
| 14 | Cardiovascular History | Description\|Past Medical History\|Disease History\|Cardiovascular History |
| 15 | Endocrine History | Description\|Past Medical History\|Disease History\|Endocrine History |
| 16 | ENT History | Description\|Past Medical History\|Disease History\|ENT History |
| 17 | Gastrointestinal History | Description\|Past Medical History\|Disease History\|Gastrointestinal History |
| 18 | Genitourinary History | Description\|Past Medical History\|Disease History\|Genitourinary History |
| 19 | Liver History | Description\|Past Medical History\|Disease History\|Liver History |
| 20 | Musculoskeletal History | Description\|Past Medical History\|Disease History\|Musculoskeletal History |
| 21 | Neurologic History | Description\|Past Medical History\|Disease History\|Neurologic History |
| 22 | OBG History | Description\|Past Medical History\|Disease History\|OB/GYN History |
| 23 | Pregnancy History | Description\|Past Medical History\|Disease History\|OB/GYN History\|Pregnancy History |
| 24 | Abnormal History of Pregnancy | Description\|Past Medical History\|Disease History\|OB/GYN History\| Pregnancy History\|Abnormal History of Pregnancy |
| 25 | Other History | Description\|Past Medical History\|Disease History\|Other History |
| 26 | Pediatrics History | Description\|Past Medical History\|Disease History\|Pediatrics History |
| 27 | Psychiatric History | Description\|Past Medical History\|Disease History\|Psychiatric History |
| 28 | Rectal History | Description\|Past Medical History\|Disease History\|Rectal History |
| 29 | Respiratory History | Description\|Past Medical History\|Disease History\|Respiratory History |
| 30 | Skin History | Description\|Past Medical History\|Disease History\|Skin History |
| 31 | Family History | Description\|Past Medical History\|Family History |
| 32 | Medical History | Description\|Past Medical History\|Medical History |
| 33 | Medication History | Description\|Past Medical History\|Medication History |
| 34 | Medication for Allergic Disorders | Description\|Past Medical History\|Medication History\|Medication for Allergic Disorders |
| 35 | Medication for Cardiovascular | Description\|Past Medical History\|Medication History\|Medication for Cardiovascular |
| 36 | Medication for CNS | Description\|Past Medical History\|Medication History\|Medication for CNS |
| 37 | Medication for Contraception | Description\|Past Medical History\|Medication History\|Medication for Contraception |
| 38 | Medication for Diagnostics | Description\|Past Medical History\|Medication History\|Medication for Diagnostics |
| 39 | Medication for Endocrine Problems | Description\|Past Medical History\|Medication History\|Medication for Endocrine Problems |
| 40 | Medication for ENT | Description\|Past Medical History\|Medication History\|Medication for ENT |
| 41 | Medication for Euthanasia | Description\|Past Medical History\|Medication History\|Medication for Euthanasia |
| 42 | Medication for Eye | Description\|Past Medical History\|Medication History\|Medication for Eye |
| 43 | Medication for GI | Description\|Past Medical History\|Medication History\|Medication for GI |
| 44 | Medication for Immune System | Description\|Past Medical History\|Medication History\|Medication for Immune System |
| 45 | Medication for Infection And Infestation | Description\|Past Medical History\|Medication History\|Medication for Infection And Infestation |
| 46 | Medication for Musculoskeletal Disorders | Description\|Past Medical History\|Medication History\|Medication for Musculoskeletal Disorders |
| 47 | Medication for Neoplastic Disorders | Description\|Past Medical History\|Medication History\|Medication for Neoplastic Disorders |
| 48 | Medication for Nutrition | Description\|Past Medical History\|Medication History\|Medication for Nutrition |
| 49 | Medication for OBGY | Description\|Past Medical History\|Medication History\|Medication for OBGY |
| 50 | Medication for Pain And Consciousness | Description\|Past Medical History\|Medication History\|Medication for Pain And Consciousness |
| 51 | Medication for Reproduction System Or Urinary System | Description\|Past Medical History\|Medication History\|Medication for Reproduction System Or Urinary System |
| 52 | Medication for Respiratory System | Description\|Past Medical History\|Medication History\|Medication for Respiratory System |
| 53 | Medication for Skin | Description\|Past Medical History\|Medication History\|Medication for Skin |
| 54 | Obstetric History | Description\|Past Medical History\|Obstetric History |
| 55 | Operation History | Description\|Past Medical History\|Operation History |
| 56 | Social History | Description\|Past Medical History\|Social History |
| 57 | Drinking History | Description\|Past Medical History\|Social History\|Drinking History |
| 58 | Smoking History | Description\|Past Medical History\|Social History\|Smoking History |
| 59 | Present Illness | Description\|Present Illness |
| 60 | Vital Signs | Description\|Vital Signs |
| 61 | Event | Even |
| 62 | Admission | Event\|Admission |
| 63 | Adverse Reaction | Event\|Adverse Reaction |
| 64 | Death | Event\|Death |
| 65 | Discharge | Event\|Discharge |
| 66 | Emergency | Event\|Emergency |
| 67 | Emergency visit | Event\|Emergency\|Emergency visit |
| 68 | Follow-up | Event\|Follow-up |
| 69 | Outpatient Care | Event\|Outpatient Care |
| 70 | Recommendation | Event\|Recommendation |
| 71 | Suicide Attempt | Event\|Suicide Attempt |
| 72 | Transfer | Event\|Transfer |
| 73 | Trauma | Event\|Trauma |
| 74 | Other Accident | Event\|Trauma\|Other Accident |
| 75 | Traffic Accident | Event\|Trauma\|Traffic Accident |
| 76 | Finding | Finding |
| 77 | Complication | Finding\|Complication |
| 78 | Current State | Finding\|Current State |
| 79 | Conscious State | Finding\|Current State\|Conscious State |
| 80 | Emotional State | Finding\|Current State\|Emotional State |
| 81 | Functional State | Finding\|Current State\|Functional State |
| 82 | Diagnosis | Finding\|Diagnosis |
| 83 | Metastasis | Finding\|Diagnosis\|Metastasis |
| 84 | Finding by Anatomic Site | Finding\|Finding by Anatomic Site |
| 85 | Finding by Image | Finding\|Finding by Image |
| 86 | General Findings | Finding\|General Findings |
| 87 | Mood | Finding\|Mood |
| 88 | Neurologic Examination | Finding\|Neurologic Examination |
| 89 | Coordination and gait | Finding\|Neurologic Examination\|Coordination and gait |
| 90 | Cranial nerves | Finding\|Neurologic Examination\|Cranial nerves |
| 91 | Mental status | Finding\|Neurologic Examination\|Mental status |
| 92 | Motor | Finding\|Neurologic Examination\|Motor |
| 93 | Reflex | Finding\|Neurologic Examination\|Reflex |
| 94 | Sensory | Finding\|Neurologic Examination\|Sensory |
| 95 | Physical Examination | Finding\|Physical Examination |
| 96 | Abdomen | Finding\|Physical Examination\|Abdomen |
| 97 | Back and extremity | Finding\|Physical Examination\|Back and extremity |
| 98 | Breast | Finding\|Physical Examination\|Breast |
| 99 | Chest | Finding\|Physical Examination\|Chest |
| 100 | Face | Finding\|Physical Examination\|Face |
| 101 | Hair | Finding\|Physical Examination\|Hair |
| 102 | Heart | Finding\|Physical Examination\|Heart |
| 103 | HEENT | Finding\|Physical Examination\|HEENT |
| 104 | Ears | Finding\|Physical Examination\|HEENT\|Ears |
| 105 | Eyes | Finding\|Physical Examination\|HEENT\|Eyes |
| 106 | Head | Finding\|Physical Examination\|HEENT\|Head |
| 107 | Nose | Finding\|Physical Examination\|HEENT\|Nose |
| 108 | Throat | Finding\|Physical Examination\|HEENT\|Throat |
| 109 | Lung | Finding\|Physical Examination\|Lung |
| 110 | Lymph node | Finding\|Physical Examination\|Lymph node |
| 111 | Mandible | Finding\|Physical Examination\|Mandible |
| 112 | Mouth | Finding\|Physical Examination\|Mouth |
| 113 | Neck | Finding\|Physical Examination\|Neck |
| 114 | Pelvic | Finding\|Physical Examination\|Pelvic |
| 115 | Spine | Finding\|Physical Examination\|Spine |
| 116 | Urinary | Finding\|Physical Examination\|Urinary |
| 117 | Vascular | Finding\|Physical Examination\|Vascular |
| 118 | Zygoma | Finding\|Physical Examination\|Zygoma |
| 119 | Problem | Finding\|Problem |
| 120 | Congenital Anomaly | Finding\|Problem\|Congenital Anomaly |
| 121 | Prognosis | Finding\|Prognosis |
| 122 | Progress | Finding\|Progress |
| 123 | Intraoperative | Finding\|Progress\|Intraoperative |
| 124 | Postoperative | Finding\|Progress\|Postoperative |
| 125 | Preoperative | Finding\|Progress\|Preoperative |
| 126 | Review of Systems | Finding\|Review of Systems |
| 127 | Sign | Finding\|Sign |
| 128 | Symptom | Finding\|Symptom |
| 129 | Objective Symptom | Finding\|Symptom\|Objective Symptom |
| 130 | Subjective Symptom | Finding\|Symptom\|Subjective Symptom |
| 131 | Test | Finding\|Test |
| 132 | Endoscopy | Finding\|Test\|Endoscopy |
| 133 | Function Test | Finding\|Test\|Function Test |
| 134 | Allergy Test | Finding\|Test\|Function Test\|Allergy Test |
| 135 | Electroencephalography | Finding\|Test\|Function Test\|Electroencephalography |
| 136 | Electromyogram | Finding\|Test\|Function Test\|Electromyogram |
| 137 | Hearing Test | Finding\|Test\|Function Test\|Hearing Test |
| 138 | Heart Function Test | Finding\|Test\|Function Test\|Heart Function Test |
| 139 | Echocardiogram | Finding\|Test\|Function Test\|Heart Function Test\|Echocardiogram |
| 140 | Electrocardiogram | Finding\|Test\|Function Test\|Heart Function Test\|Electrocardiogram |
| 141 | Holter Monitoring | Finding\|Test\|Function Test\|Heart Function Test\|Holter Monitoring |
| 142 | Polysomnography | Finding\|Test\|Function Test\|Polysomnography |
| 143 | Pulmonary Function Test | Finding\|Test\|Function Test\|Pulmonary Function Test |
| 144 | Laboratory Tests | Finding\|Test\|Laboratory Tests |
| 145 | Nuclear Medicine | Finding\|Test\|Nuclear Medicine |
| 146 | Gallium Scan | Finding\|Test\|Nuclear Medicine\|Gallium Scan |
| 147 | MIBG | Finding\|Test\|Nuclear Medicine\|MIBG |
| 148 | PET/CT Scan | Finding\|Test\|Nuclear Medicine\|PET/CT Scan |
| 149 | Pathologic Findings | Finding\|Test\|Pathologic Findings |
| 150 | Autopsy | Finding\|Test\|Pathologic Findings\|Autopsy |
| 151 | Cytology | Finding\|Test\|Pathologic Findings\|Cytology |
| 152 | Molecular Pathology | Finding\|Test\|Pathologic Findings\|Molecular Pathology |
| 153 | Surgical Pathology | Finding\|Test\|Pathologic Findings\|Surgical Pathology |
| 154 | Radiology | Finding\|Test\|Radiology |
| 155 | CT | Finding\|Test\|Radiology\|CT |
| 156 | MRI | Finding\|Test\|Radiology\|MRI |
| 157 | Ultrasonography | Finding\|Test\|Radiology\|Ultrasonography |
| 158 | Procedure | Procedure |
| 159 | Anesthesia Procedure | Procedure\|Anesthesia Procedure |
| 160 | Cardiopulmonary Resuscitation | Procedure\|Cardiopulmonary Resuscitation |
| 161 | Dialysis Procedure | Procedure\|Dialysis Procedure |
| 162 | Hemodialysis | Procedure\|Dialysis Procedure\|Hemodialysis |
| 163 | Peritoneal Dialysis | Procedure\|Dialysis Procedure\|Peritoneal Dialysis |
| 164 | Diet | Procedure\|Diet |
| 165 | NPO | Procedure\|Diet\|NPO |
| 166 | Dressing patients | Procedure\|Dressing patients |
| 167 | Education | Procedure\|Education |
| 168 | Medical Consult | Procedure\|Medical Consult |
| 169 | Plan | Procedure\|Plan |
| 170 | Care Goal | Procedure\|Plan\|Care Goal |
| 171 | Care Plan | Procedure\|Plan\|Care Plan |
| 172 | Education Plan | Procedure\|Plan\|Education Plan |
| 173 | Prescription Procedure | Procedure\|Prescription Procedure |
| 174 | Antineoplastic Drug/Agent Therapy | Procedure\|Prescription Procedure\|Antineoplastic Drug/Agent Therapy |
| 175 | Chemotherapy | Procedure\|Prescription Procedure\|Chemotherapy |
| 176 | Current medication | Procedure\|Prescription Procedure\|Current medication |
| 177 | Hormone Therapy | Procedure\|Prescription Procedure\|Hormone Therapy |
| 178 | Physiotherapy | Procedure\|Prescription Procedure\|Physiotherapy |
| 179 | Language Therapy | Procedure\|Prescription Procedure\|Physiotherapy\|Language Therapy |
| 180 | Occupational Therapy | Procedure\|Prescription Procedure\|Physiotherapy\|Occupational Therapy |
| 181 | Psychotherapy | Procedure\|Prescription Procedure\|Physiotherapy\|Psychotherapy |
| 182 | Rehabilitation Therapy | Procedure\|Prescription Procedure\|Physiotherapy\|Rehabilitation Therapy |
| 183 | Radiotherapy | Procedure\|Prescription Procedure\|Radiotherapy |
| 184 | Surgery | Procedure\|Surgery |
| 185 | Tissue extracts | Procedure\|Tissue extracts |
| 186 | Transfusion | Procedure\|Transfusion |
| 187 | Transplantation | Procedure\|Transplantation |
| 188 | Treatment | Procedure\|Treatment |

**Supplementary Table S3.** Full names of definition sources. In form type, HL7 and CDA are represented 6 HL7 templates and 25 clinical documents from 5 teaching hospitals, respectively.

| **Coverage**  **Type** | **Form**  **Type** | **CMDO concept (Level)** | **CDE Name** |
| --- | --- | --- | --- |
| *Too broad* | HL7 (n=14) | Description (1^st^) | Assessment_and_Plan_Description |
|  |  | Description (1^st^) | Condition_Status_Specify |
|  |  | Description (1^st^) | Condition_Type_Specify |
|  |  | Description (1^st^) | General_Status_Specify |
|  |  | Description (1^st^) | Hospital_Discharge_Physical_Status_Specify |
|  |  | Description (1^st^) | Reason_for_Referral_Specify |
|  |  | Finding (1^st^) | Objective_Finding_Specify |
|  |  | Procedure (1^st^) | Operative_Note_Fluids_Specify |
|  |  | Procedure (1^st^) | Patient_Disposition_Specify |
|  |  | Procedure (1^st^) | Procedure_Estimated_Blood_Loss_Specify |
|  |  | Procedure (1^st^) | Procedure_Implants_Occur_Specify |
|  |  | Procedure (1^st^) | Procedure_Location_Specify |
|  |  | Procedure (1^st^) | Procedure_Specimens_Taken_Name_Specify |
|  |  | Procedure (1^st^) | Specimens_Removed_Specify |
|  | CDA (n=15) | Procedure (1^st^) | Activated_Charcoal_Type_Specify |
|  |  | Procedure (1^st^) | Extracorporeal_Treatment_Type_Category |
|  |  | Procedure (1^st^) | Gastric_Decontamination_Type_Category |
|  |  | Procedure (1^st^) | Gastric_Lavage_Via_Category |
|  |  | Procedure (1^st^) | Gastric_Lavage_Volume |
|  |  | Procedure (1^st^) | Hemoperfusion_Performed_Specify |
|  |  | Treatment (1^st^) | Continuous_Renal_Replacement Therapy_Setting_Specify |
|  |  | Test (2^nd^) | Intraoperative_Sended_to_Laboratory_Culture_Yes_or_No_Indicator |
|  |  | Test (2^nd^) | Intraoperative_Sended_to_laboratory_Cytology_Yes_or_No_Indicator |
|  |  | Test (2^nd^) | Intraoperative_Sended_to_Laboratory_Specimen_Yes_or_No_Indicator |
|  |  | Surgery (2^nd^) | Skin_Incision_Specify |
|  |  | Transfer (2^nd^) | When_Transfer_Discharge_Other_Preparation_Specify |
|  |  | Transfer (2^nd^) | When_Transfer_Discharge_Preparation Items_Type_Category |
|  |  | Transfer (2^nd^) | When_Transfer_Discharge_Transfer_Hospital_Selection_ |
|  |  | Transfer (2^nd^) | When_Transfer_Discharge_Transfer_Reason_Specify |
| *Too specific* | HL7 (n=4) | Functional State (3^rd^) | Functional_Condition_Status |
|  |  | Functional State (3^rd^) | Functional_Condition_Type_Specify |
|  |  | Functional State (3^rd^) | Functional_Condition_Effective_Dates |
|  |  | Subjective Symptom (3^rd^) | Subjective_Symptom_Specify |
|  | CDA  (n=7) | Care Goal (3^rd^) | Goal_of_Care_Plan_Specify |
|  |  | Care Plan (3^rd^) | Comment_of_care_plan_Specify |
|  |  | Conscious State (3^rd^) | Consciousness_State_of_Patient_Specify |
|  |  | Congenital Anomaly (4^th^) | Congenital_Anomaly_Specify |
|  |  | Congenital Anomaly (4^th^) | Congenital_Anomaly_None_Indicator |
|  |  | Education Plan (3^rd^) | Content_of_Education_Plan_Specify |
|  |  | Electrocardiogram (5^th^) | Electrocardiogram_Specify |

**Supplementary Table S4.** List of CDEs matched to CMDO concepts from two source data. In form type, HL7 and CDA are represented 6 HL7 templates and 25 clinical documents from 5 teaching hospitals, respectively.

| **Form**  **Type** | **First-level Class** | **CMDO Level** | **Matched CMDO concept** | **CDE Name** |
| --- | --- | --- | --- | --- |
| HL7 | Description | 1 | Description | Objective Finding Specify |
| HL7 | Description | 1 | Description | Reason for Referral Specify |
| HL7 | Description | 1 | * Description^Admission | Hospital Course Specify |
| HL7 | Description | 2 | *Adverse reaction^  Alerts^Allergy | Allergies, Adverse Reactions, Alerts Induced Substance Specify |
| HL7 | Description | 2 | *Adverse reaction^  Alerts^Allergy | Allergies, Adverse Reactions, Alerts Reaction Specify |
| HL7 | Description | 2 | *Adverse reaction^  Alerts^Allergy | Allergies, Adverse Reactions, Alerts Status Specify |
| HL7 | Description | 2 | *Assessment^Plan | Assessment and Plan Description |
| HL7 | Description | 2 | Chief Complaint | Chief Complaint Specify |
| HL7 | Description | 2 | General findings | Condition Status Specify |
| HL7 | Description | 2 | General findings | Condition Type Specify |
| HL7 | Description | 2 | General findings | General Status Specify |
| HL7 | Description | 2 | General findings | Physical Examination General Status |
| HL7 | Description | 2 | General findings | Problem Effective Dates |
| HL7 | Description | 2 | Past History | Medical History |
| HL7 | Description | 2 | Past History | Past Medical History |
| HL7 | Description | 2 | Present illness | History of Present Illness Specify |
| HL7 | Description | 2 | Immunization | Immunizations Status Specify |
| HL7 | Description | 2 | Immunization | Vaccine Date Specify |
| HL7 | Description | 2 | Immunization | Vaccine Type Specify |
| HL7 | Description | 2 | Vital Signs | Blood Pressure of Vital Sign Measurement |
| HL7 | Description | 2 | Vital Signs | Vital Sign Measurement |
| HL7 | Description | 3 | Family History | Family History Specify |
| HL7 | Description | 3 | Family History | Family History Diagnosis Specify |
| HL7 | Description | 3 | Family History | Family History Relation Specify |
| HL7 | Description | 3 | Family History | Family History Status Specify |
| HL7 | Description | 3 | Family History | Family History Age At Onset |
| HL7 | Description | 3 | Medication History | Medications Comments |
| HL7 | Description | 3 | Medication History | Medications History |
| HL7 | Description | 3 | Medication History | Medications Name and Usage Specify |
| HL7 | Description | 3 | Operation History | Past Surgical History |
| HL7 | Description | 3 | Social History | Social History |
| HL7 | Description | 3 | Social History | Social History Description |
| HL7 | Description | 3 | Social History | Social History Element |
| HL7 | Description | 3 | Social History | Social History Effective Dates |
| HL7 | Description | 4 | *Adverse reaction^Allergy | Allergies Type Specify |
| HL7 | Description | 4 | *Adverse reaction^Allergy | Allergies and Adverse Reaction Description |
| HL7 | Description | 4 | *Adverse reaction^  Alerts^Allergy | Allergies, Adverse Reactions, Alerts Induced Substance Specify |
| HL7 | Description | 4 | *Adverse reaction^  Alerts^Allergy | Allergies, Adverse Reactions, Alerts Reaction Specify |
| HL7 | Description | 4 | *Adverse reaction^  Alerts^Allergy | Allergies, Adverse Reactions, Alerts Status Specify |
| HL7 | Event | 2 | * Description^Admission | Hospital Course Specify |
| HL7 | Event | 2 | *Adverse reaction^Allergy | Allergies Type Specify |
| HL7 | Event | 2 | *Adverse reaction^Allergy | Allergies and Adverse Reaction Description |
| HL7 | Event | 2 | *Adverse reaction^  Alerts^Allergy | Allergies, Adverse Reactions, Alerts Induced Substance Specify |
| HL7 | Event | 2 | *Adverse reaction^  Alerts^Allergy | Allergies, Adverse Reactions, Alerts Reaction Specify |
| HL7 | Event | 2 | *Adverse reaction^  Alerts^Allergy | Allergies, Adverse Reactions, Alerts Status Specify |
| HL7 | Event | 2 | *Care Plan^Follow-up | Plan of Care Planned Activity |
| HL7 | Event | 2 | *Diet^Discharge | Discharge Diet Type Specify |
| HL7 | Event | 2 | *Functional State^Discharge | Functional Condition Status |
| HL7 | Event | 2 | *Functional State^Discharge | Functional Condition Effective Dates |
| HL7 | Event | 2 | *Functional State^Discharge | Functional Condition Type Specify |
| HL7 | Event | 2 | *Diagnosis^Discharge | Hospital Discharge Diagnosis Name |
| HL7 | Event | 2 | *Diagnosis^Discharge | Hospital Discharge Diagnosis Date |
| HL7 | Event | 2 | *Physical examination^Discharge | Hospital Discharge Physical Status Specify |
| HL7 | Event | 2 | *Prescription procedure^Discharge | Hospital Discharge Medicine Instruction Specify |
| HL7 | Event | 2 | *Prescription procedure^Discharge | Hospital Discharge Medicine Name Specify |
| HL7 | Finding | 1 | *Finding^Surgery | Operative Note Findings Specify |
| HL7 | Finding | 2 | Test Result | Exam Result Specify |
| HL7 | Finding | 2 | Test Result | Other Tests Type Specify |
| HL7 | Finding | 2 | *Diagnosis^Discharge | Hospital Discharge Diagnosis Name |
| HL7 | Finding | 2 | *Diagnosis^Discharge | Hospital Discharge Diagnosis Date |
| HL7 | Finding | 2 | Review of Systems | Review of Systems Condition Specify |
| HL7 | Finding | 2 | *Test Result^Treatment | Treatment Result Specify |
| HL7 | Finding | 2 | *Complication^Surgery | Complications Occurrence Specify |
| HL7 | Finding | 2 | Neurologic Examination | Physical Examination Neurologic System Result |
| HL7 | Finding | 2 | *Diagnosis^Surgery | Postprocedure Diagnosis Specify |
| HL7 | Finding | 2 | *Diagnosis^Surgery | Postoperative Diagnosis Specify |
| HL7 | Finding | 2 | *Diagnosis^Surgery | Preoperative Diagnosis Specify |
| HL7 | Finding | 2 | Problem | Finding Problem Specify |
| HL7 | Finding | 3 | Laboratory Test | Laboratory Information Measurement |
| HL7 | Finding | 3 | Laboratory Test | Laboratory Information, Chemistries and Drug Levels |
| HL7 | Finding | 3 | Laboratory Test | Liver Functions and Other Laboratory Values |
| HL7 | Finding | 3 | PE of Abdomen | Physical Examination Abdomen Result |
| HL7 | Finding | 3 | PE of Back and extremity | Physical Examination Back Result |
| HL7 | Finding | 3 | PE of Back and extremity | Physical Examination Extremities Result |
| HL7 | Finding | 3 | PE of HEENT | Physical Examination HEENT(Head,Ear,Eye,Nose,Throat) Result |
| HL7 | Finding | 3 | PE of Chest | Physical Examination Thorax Result |
| HL7 | Finding | 3 | PE of Heart | Physical Examination Heart Result |
| HL7 | Finding | 3 | Subjective Symptom | Subjective Symptom Specify |
| HL7 | Finding | 5 | Electrocardiogram | Electrocardiogram (EKG) Information |
| HL7 | Procedure | 1 | Procedure | Patient Disposition Specify |
| HL7 | Procedure | 1 | Procedure | Planned Procedure Name Specify |
| HL7 | Procedure | 1 | Procedure | Procedure Date Specify |
| HL7 | Procedure | 1 | Procedure | Procedure Description |
| HL7 | Procedure | 1 | Procedure | Procedure Estimated Blood Loss Specify |
| HL7 | Procedure | 1 | Procedure | Procedure Finding Specify |
| HL7 | Procedure | 1 | Procedure | Procedure Implants Occur Specify |
| HL7 | Procedure | 1 | Procedure | Procedure Indications Specify |
| HL7 | Procedure | 1 | Procedure | Procedure Location Specify |
| HL7 | Procedure | 1 | Procedure | Procedure Name Specify |
| HL7 | Procedure | 1 | Procedure | Procedure Specimens Taken Name Specify |
| HL7 | Procedure | 2 | Anesthesia procedures | Anesthesia Type Specify |
| HL7 | Procedure | 2 | Physical Examination | Height of Nutritional Evaluation Measurement |
| HL7 | Procedure | 2 | *Prescription procedure^Discharge | Hospital Discharge Medicine Instruction Specify |
| HL7 | Procedure | 2 | *Prescription procedure^Discharge | Hospital Discharge Medicine Name Specify |
| HL7 | Procedure | 2 | *Physical examination^Discharge | Hospital Discharge Physical Status Specify |
| HL7 | Procedure | 2 | Prescription procedure | Medications Administered Specify |
| HL7 | Procedure | 2 | *Prognosis^Surgery | Operative Indications Description |
| HL7 | Procedure | 2 | *Finding^Surgery | Operative Note Findings Specify |
| HL7 | Procedure | 2 | Surgery | Operative Note Fluids Specify |
| HL7 | Procedure | 2 | Surgery | Patient Disposition Specify |
| HL7 | Procedure | 2 | Physical Examination | Physical Examination Finding Specify |
| HL7 | Procedure | 2 | *Plan^Surgery | Postoperative Plan Specify |
| HL7 | Procedure | 2 | *Diagnosis^Surgery | Postprocedure Diagnosis Specify |
| HL7 | Procedure | 2 | *Plan^Surgery | Preoperative Plan Specify |
| HL7 | Procedure | 2 | *Diagnosis^Surgery | Preoperative Diagnosis Specify |
| HL7 | Procedure | 2 | Surgery | Specimens Removed Specify |
| HL7 | Procedure | 2 | Surgery | Surgery Description |
| HL7 | Procedure | 2 | Surgery | Surgical Drains Specify |
| HL7 | Procedure | 2 | Surgery | Surgical Implants Specify |
| HL7 | Procedure | 2 | Surgery | Surgical Procedure Specify |
| HL7 | Procedure | 2 | Physical Examination | Weight of Nutritional Evaluation Measurement |
| HL7 | Procedure | 3 | Care Plan | Plan of Care Planned Date |
| HL7 | Procedure | 3 | *Care Plan^Follow-up | Plan of Care Planned Activity |
| CDA | Description | 1 | *Descripton^Death | Cause of Death Specify |
| CDA | Description | 1 | *Descripton^Emergency | Hospital Arrival Patient Status Category |
| CDA | Description | 2 | Assessment | Impression Specify |
| CDA | Description | 2 | Assessment | Risk Evaluation Specify |
| CDA | Description | 2 | Assessment | Pain Evaluation Specify |
| CDA | Description | 2 | Chief Complaint | Chief Complaint Specify |
| CDA | Description | 2 | Chief Complaint | Chief Symptom and Reason Coming to Help of Chief Complain Specify |
| CDA | Description | 2 | Chief Complaint | Duration of Chief Complain Specify |
| CDA | Description | 2 | Chief Complaint | Others of Chief Complain |
| CDA | Description | 2 | Chief Complaint | Onset of Chief Complain |
| CDA | Description | 2 | Demographics | Address Description |
| CDA | Description | 2 | Demographics | Attending Physician Name Specify |
| CDA | Description | 2 | Demographics | Cell Phone Number |
| CDA | Description | 2 | Demographics | Commission Doctor Name Specify |
| CDA | Description | 2 | Demographics | Name of Duty Doctor Specify |
| CDA | Description | 2 | Demographics | Home Telephone Number |
| CDA | Description | 2 | Demographics | Name of Recording Doctor |
| CDA | Description | 2 | Demographics | Patient Age Value |
| CDA | Description | 2 | Demographics | Patient Name |
| CDA | Description | 2 | Demographics | Patient Number |
| CDA | Description | 2 | Demographics | Patient Sex Type |
| CDA | Description | 2 | Demographics | Patient Type Category |
| CDA | Description | 2 | Demographics | Patient Ward |
| CDA | Description | 2 | Demographics | Phone Number |
| CDA | Description | 2 | Present Illness | PI(Present Illness) Specify |
| CDA | Description | 2 | Demographics | Patient Private Ward |
| CDA | Description | 2 | Demographics | Protector Address Specify |
| CDA | Description | 2 | Demographics | Protector Name Specify |
| CDA | Description | 2 | Demographics | Clinical Document Recording Date |
| CDA | Description | 2 | Demographics | Patient Registration Date Specify |
| CDA | Description | 2 | Demographics | Relationship With Patient |
| CDA | Description | 2 | Demographics | Serial Number Specify |
| CDA | Description | 2 | Demographics | Treatment Department Specify |
| CDA | Description | 2 | Demographics | Assistant Surgeon's Name Specify |
| CDA | Description | 2 | *Demographics^Discharge | Discharge During Charge Type Category |
| CDA | Description | 2 | *Demographics^Emergency | Name of Emergency Doctor Specify |
| CDA | Description | 2 | Anesthesia procedures^ *Demographics | Anesthesia Doctor's Name Specify |
| CDA | Description | 2 | Demographics | Assistant Surgeon's Name Specify |
| CDA | Description | 2 | Surgery^*Demographics | Nursing Name Specify |
| CDA | Description | 2 | Surgery^*Demographics | Surgeon's Name Specify |
| CDA | Description | 2 | Present Illness | Information provider of PI(Present Illness) Specify |
| CDA | Description | 2 | Present Illness | Resident Registration Number of Patient |
| CDA | Description | 2 | *OBYGN^Present Illness | Term Infant Type Category |
| CDA | Description | 2 | Past History | Other Disease Specify |
| CDA | Description | 2 | Past History | Past History Description |
| CDA | Description | 2 | Past History | Past History Type Category |
| CDA | Description | 2 | Past History | Past Medical History Tumor Occurrence Specify |
| CDA | Description | 2 | Past History | Past Medical History Trauma History Specify |
| CDA | Description | 2 | Immunization | Vaccination Performed Indicator |
| CDA | Description | 2 | Immunization | Vaccination Specify |
| CDA | Description | 2 | Immunization | Vaccination Description |
| CDA | Description | 3 | Obstetric History | Para Specify |
| CDA | Description | 3 | *Family History^Diagnosis | Infant and Adolescent Family Relations Type Category |
| CDA | Description | 3 | *Social History^Diagnosis | The Old and Infirm Dressing Type Category |
| CDA | Description | 3 | *Medication History ^Admission | Be in Hospital Medication Specify |
| CDA | Description | 3 | Social History | Before Disease Occur Personality |
| CDA | Description | 3 | Social History | Birth History Description |
| CDA | Description | 3 | * Medication History ^Discharge | Discharge Medicine Yes or No Indicator |
| CDA | Description | 3 | Family History | Family History Type Category |
| CDA | Description | 3 | Family History | Family History Description |
| CDA | Description | 3 | *Medication History^Present Illness | HAP(Hospital-acquired Pneumonia), Yes or No Indicator |
| CDA | Description | 3 | *Disease History^Function Test | Infectious Patient Type Category |
| CDA | Description | 3 | Payor | Medical Insurance Specify |
| CDA | Description | 3 | Medication History | Medication Specify |
| CDA | Description | 3 | Social History | Occupation Type Category |
| CDA | Description | 3 | *Medication History^Transfer | Other Hospital Antibiotic Name |
| CDA | Description | 3 | *Medication History^Transfer | Other Hospital Antibiotic Use Period |
| CDA | Description | 3 | *Medication History^Transfer | Other Hospital Antibiotic Use, Yes or No Indicator |
| CDA | Description | 3 | Medication History | Other Take Medicine Specify |
| CDA | Description | 3 | Obstetric History | Parity History Description |
| CDA | Description | 3 | Operation History | Past Medical History Operation History Specify |
| CDA | Description | 3 | *Admission^Medical History | Admission Reason and Medical History Summary |
| CDA | Description | 3 | Admission^*Operation History | Admission and Operation History Yes or No Indicator |
| CDA | Description | 3 | Admission^*Operation History | Admission and Operation History Specify |
| CDA | Description | 3 | Admission^*Operation History | Admission and Operation Time Specify |
| CDA | Description | 3 | Social History | Patients Friendship of Adolescent Type Category |
| CDA | Description | 3 | *Physical Examination^Developmental History | Physical Examination Nutritional and Development Status Category |
| CDA | Description | 3 | Social History | Pregnant and Lactating Yes or Indicator |
| CDA | Description | 3 | Developmental History | Secondary Sexual Character of Adolescent Type Category |
| CDA | Description | 3 | Social History | Social History Type |
| CDA | Description | 3 | Social History | Social History Description |
| CDA | Description | 3 | Social History | Social History Marital Status |
| CDA | Description | 3 | Social History | Social History Occupation Name |
| CDA | Description | 3 | Social History | Previous Scabies Patient Cohabitation, Yes or No Indicator |
| CDA | Description | 3 | Medication History | Take Medicine of the Origin Specify |
| CDA | Description | 3 | Medication History | Take Medicine Yes or No indicator |
| CDA | Description | 3 | Medication History | Taking Medicine Specify |
| CDA | Description | 3 | *Social History^Diagnosis | The Old and Infirm Bathing Type Category |
| CDA | Description | 3 | *Social History^Diagnosis | The Old and Infirm Meal Type Category |
| CDA | Description | 3 | *Social History^Diagnosis | The Old and Infirm Movement Type Category |
| CDA | Description | 3 | *Social History^Diagnosis | The Old and Infirm Toilet Use Type Category |
| CDA | Description | 3 | *Social History^Diagnosis | The Old and Infirm Urine and Feces Control Type Category |
| CDA | Description | 4 | Allergy | Past Allergy Specify |
| CDA | Description | 4 | Allergy | Past History Allergy Type Category |
| CDA | Description | 4 | Allergy | Past History Other Allergy Specify |
| CDA | Description | 4 | *Allergy^Other History | Past Medical History Food and Other Allergy, Present or Absent Indicator |
| CDA | Description | 4 | *Allergy^Diet | Past History Food Allergy Specify |
| CDA | Description | 4 | Drinking History | Alcohol History Specify |
| CDA | Description | 4 | Drinking History | Drinking Capacity Specify |
| CDA | Description | 4 | Drinking History | Drinking Frequency Specify |
| CDA | Description | 4 | Drinking History | Drinking History Type Category |
| CDA | Description | 4 | Drinking History | Drinking History Alcohol Type Category |
| CDA | Description | 4 | Drinking History | Drinking History Month Frequency |
| CDA | Description | 4 | Drinking History | Drinking History Once Drinking Capacity |
| CDA | Description | 4 | Drinking History | Drinking History Period |
| CDA | Description | 4 | Endocrine History^*HEENT | Parathyroid Hormone Status Specify |
| CDA | Description | 4 | Neurologic History | Diplopia, Plus or Minus Indicator |
| CDA | Description | 4 | Neurologic History | Dysarthria, Plus or Minus Indicator |
| CDA | Description | 4 | Neurologic History | Dysphagia, Plus or Minus Indicator |
| CDA | Description | 4 | Neurologic History | Limitation of EOM(Extraocular Movement), Plus or Minus Indicator |
| CDA | Description | 4 | Neurologic History | Neurologic Abnormal Specify |
| CDA | Description | 4 | Neurologic History | Nystagmus Occurrence, Plus or Minus Indicator |
| CDA | Description | 4 | Neurologic History | Optic Atrophy Location Category |
| CDA | Description | 4 | Medication for Allergic Disorders | Past History Drug Allergy Specify |
| CDA | Description | 4 | Medication for Allergic Disorders | Past Medical History Antibiotics allergy Type Category |
| CDA | Description | 4 | OBYGN History | Congenital Anomaly Specify |
| CDA | Description | 4 | OBYGN History | Congenital Anomaly, None Indicator |
| CDA | Description | 4 | OBYGN History | Delivery Type Category |
| CDA | Description | 4 | OBYGN History | Perinatal Problem Specify |
| CDA | Description | 4 | OBYGN History | Perinatal Problem, None Indicator |
| CDA | Description | 4 | *OBYGN^Present Illness | Term Infant Type Category |
| CDA | Description | 4 | Review of Systems^*Respiratory History | Respiratory Status Specify |
| CDA | Description | 4 | Review of Systems^*Respiratory History | Respiratory Type Category |
| CDA | Description | 4 | Pediatrics History | Other Pediatric History Specify |
| CDA | Description | 4 | Psychiatric History | Psychological Disease Specify |
| CDA | Description | 4 | Smoking History | Quit Smoking Period |
| CDA | Description | 4 | Smoking History | Smoking Specify |
| CDA | Description | 4 | Smoking History | Smoking History Per Day Cigarettes Consumption Count |
| CDA | Description | 4 | Smoking History | Smoking History Smoking Period (Year) |
| CDA | Description | 4 | Smoking History | Smoking History Type Category |
| CDA | Description | 4 | *Symptom^Skin | Pruritus, Skin Rash, Yes or No Indicator |
| CDA | Description | 5 | Abnormal History of Mother Pregnancy | Mother Abnormality Indicator |
| CDA | Description | 5 | Abnormal History of Mother Pregnancy | Mother Abnormality Specify |
| CDA | Event | 2 | *Descripton^Death | Cause of Death Specify |
| CDA | Event | 2 | *Descripton^Emergency | Hospital Arrival Patient Status Category |
| CDA | Event | 2 | *Demographics^Discharge | Discharge During Charge Type Category |
| CDA | Event | 2 | *Demographics^Emergency | Name of Emergency Doctor Specify |
| CDA | Event | 2 | *Medication History ^Admission | Be in Hospital Medication Specify |
| CDA | Event | 2 | * Medication History ^Discharge | Discharge Medicine Yes or No Indicator |
| CDA | Event | 2 | Outpatient Clinic | Hospital Care Date |
| CDA | Event | 2 | *Treatment^Outpatient Clinic | Outpatient Treatment Test Date Specify |
| CDA | Event | 2 | *Physical Examination^ Outpatient Clinic | The First Medical Examination Date Specify |
| CDA | Event | 2 | Admission^*Operation History | Admission and Operation History Yes or No Indicator |
| CDA | Event | 2 | Admission^*Operation History | Admission and Operation History Specify |
| CDA | Event | 2 | Admission^*Operation History | Admission and Operation Time Specify |
| CDA | Event | 2 | Admission | Admission Course Specify |
| CDA | Event | 2 | Admission | Admission Date |
| CDA | Event | 2 | Admission | Admission Department Specify |
| CDA | Event | 2 | *Admission^Progress | Admission Progress |
| CDA | Event | 2 | *Admission^Medical History | Admission Reason and Medical History Summary |
| CDA | Event | 2 | *Symptom^Admission | Pain Yes or No Indicator |
| CDA | Event | 2 | Admission | The Number of Times of Admission Specify |
| CDA | Event | 2 | Admission | HOD(Hospitalization day) Specify |
| CDA | Event | 2 | Admission | Hospital Admission Route Category |
| CDA | Event | 2 | Follow-up | Appointed Day Specify |
| CDA | Event | 2 | Discharge | Consciousness State of Patient Specify |
| CDA | Event | 2 | Discharge | Discharge Criteria Specify |
| CDA | Event | 2 | Discharge | Discharge Date |
| CDA | Event | 2 | Discharge | Discharge Department |
| CDA | Event | 2 | Discharge | Discharge Method Specify |
| CDA | Event | 2 | *Discharge^Plan | Discharge Plan Description |
| CDA | Event | 2 | Discharge | Discharge Status Specify |
| CDA | Event | 2 | Discharge | Discharge Type Specify |
| CDA | Event | 2 | Discharge | Disease Onset Day and Time Specify |
| CDA | Event | 2 | Discharge | Hospital Discharge Time Category |
| CDA | Event | 2 | Discharge | Remarks of Patient Specify |
| CDA | Event | 2 | Discharge | The Sphere of Activity Specify |
| CDA | Event | 2 | Discharge^*Diet | Dietetic Theraphy Specify |
| CDA | Event | 2 | Discharge^*Prescription procedure | Discharge Medicine Dose Period Specify |
| CDA | Event | 2 | Discharge^*Prescription procedure | Discharge Medicine Name Specify |
| CDA | Event | 2 | Discharge^*Prescription procedure | Discharge Medicine Usage Specify |
| CDA | Event | 2 | Discharge^*Prescription procedure | Medication Dose Frequency |
| CDA | Event | 2 | Discharge^*Prescription procedure | Medication Quantity |
| CDA | Event | 2 | Discharge^*Prescription procedure | Medication Unit |
| CDA | Event | 2 | Emergency | Location of Patient Specify |
| CDA | Event | 2 | Emergency | Nursing Record |
| CDA | Event | 2 | Emergency | Occurrence Course of Patient Specify |
| CDA | Event | 2 | Emergency | Occurrence Time of Patient Specify |
| CDA | Event | 2 | *Outpatient Clinic ^Emergency Visit | Come to Help Information Specify |
| CDA | Event | 2 | Treatment^Discharge | Treatment Result Specify |
| CDA | Event | 2 | *Treatment^Outpatient Clinic | Outpatient Treatment Test Date Specify |
| CDA | Event | 2 | Follow-up | F/U Attended Matters Specify |
| CDA | Event | 2 | *Follow-up^Care Plan | F/U Care Plan Associated Comment Specify |
| CDA | Event | 2 | *Follow-up^Plan | F/U Plan Specify |
| CDA | Event | 2 | *Medication History^Transfer | Other Hospital Antibiotic Name |
| CDA | Event | 2 | *Medication History^Transfer | Other Hospital Antibiotic Use Period |
| CDA | Event | 2 | *Medication History^Transfer | Other Hospital Antibiotic Use, Yes or No Indicator |
| CDA | Event | 2 | Transfer | Other Hospital Data Type Category |
| CDA | Event | 2 | Transfer | Other Hospital Data, Present or Absent Indicator |
| CDA | Event | 2 | Transfer | Transfer Date |
| CDA | Event | 2 | Transfer | Transfer Department |
| CDA | Event | 2 | Transfer | Transfer During Need Treatment Specify |
| CDA | Event | 2 | Transfer | Transferred from Department Date |
| CDA | Event | 2 | Transfer | Transferred from Department Physician Name |
| CDA | Event | 2 | Transfer | Transferred from Hospital Type Category |
| CDA | Event | 2 | Transfer | When Transfer Discharge Drug Preparations Specify |
| CDA | Event | 2 | Transfer | When Transfer Discharge Hospital Name and Contacted Physician Specify |
| CDA | Event | 2 | Transfer | When Transfer Discharge Other Preparation Specify |
| CDA | Event | 2 | Transfer | When Transfer Discharge Preparation Items Type Category |
| CDA | Event | 2 | Transfer | When Transfer Discharge Transfer Hospital Selection Method Specify |
| CDA | Event | 3 | Emergency visit | Emergency Room Arrival Time |
| CDA | Event | 3 | Emergency visit | Emergency Room Visit Method Category |
| CDA | Event | 3 | *Outpatient Clinic ^Emergency Visit | Come to Help Information Specify |
| CDA | Finding | 1 | Finding | Examination Finding Nonspecific Specify |
| CDA | Finding | 1 | Finding | Examination Finding Specify |
| CDA | Finding | 2 | Physical examination | Abnormal of Head and Neck Specify |
| CDA | Finding | 2 | Test Results | Additive of Toxic Element Specify |
| CDA | Finding | 2 | Review of Systems | Allergic & Rheumatologic Type Category |
| CDA | Finding | 2 | Test Results | Ankle Clonus Location Type |
| CDA | Finding | 2 | Test Results | Babinski Sign Location Type |
| CDA | Finding | 2 | Physical examination | Blood Type Category |
| CDA | Finding | 2 | Physical examination | Blood Yes or No indicator |
| CDA | Finding | 2 | Physical examination | Blue-dot sign Yes or No indicator |
| CDA | Finding | 2 | Physical examination | BMI(Body Mass Index) of Nutritional Evaluation Value |
| CDA | Finding | 2 | Physical examination | Cremasteric reflex Yes or No indicator |
| CDA | Finding | 2 | *Physical Examination^ Outpatient Clinic | The First Medical Examination Date Specify |
| CDA | Finding | 2 | Review of Systems^*Cardiovascular | Cardiovascular Status Specify |
| CDA | Finding | 2 | Review of Systems^*Cardiovascular | Cardiovascular Type Category |
| CDA | Finding | 2 | Test Results | Cerebellar Function Test Result, No Specific Finding Indicator |
| CDA | Finding | 2 | Diagnosis | Chief Diagnosis Name of Postoperative Specify |
| CDA | Finding | 2 | Diagnosis | Chief Diagnosis Name Specify |
| CDA | Finding | 2 | Diagnosis | Chief Diagnosis Name of Preoperative Specify |
| CDA | Finding | 2 | Physical examination | Dental Caries Yes or No Indicator |
| CDA | Finding | 2 | Physical examination | Denture Yes or No Indicator |
| CDA | Finding | 2 | Physical examination | Developmental Anomalies None Indicator |
| CDA | Finding | 2 | Diagnosis | Diagnosis Name Specify |
| CDA | Finding | 2 | Diagnosis | Diagnosis Name of Postoperative Specify |
| CDA | Finding | 2 | Diagnosis | Diagnosis Name of Preoperative Specify |
| CDA | Finding | 2 | Test Results | Examination Finding Type Category |
| CDA | Finding | 2 | Test Results | Exposure Volume of Toxic Element Specify |
| CDA | Finding | 2 | Diagnosis | Final Additional Diagnosis Name |
| CDA | Finding | 2 | Diagnosis | Final Principal Diagnosis Name |
| CDA | Finding | 2 | Test Results | Frequency of Pain Evaluation Specify |
| CDA | Finding | 2 | Physical examination | Fontanelle Status Specify |
| CDA | Finding | 2 | Physical examination | General Appearance Type Category |
| CDA | Finding | 2 | Physical examination | General Survey Appearance Description |
| CDA | Finding | 2 | Physical examination | Genitourinary Examination Other Specify |
| CDA | Finding | 2 | Physical examination | Genitourinary Result |
| CDA | Finding | 2 | Physical examination | Growth Retardation, None Indicator |
| CDA | Finding | 2 | Review of Systems^*Gastrointestinal | Gastrointestinal Status Specify |
| CDA | Finding | 2 | Review of Systems^*Gastrointestinal | Gastrointestinal Type Category |
| CDA | Finding | 2 | General findings | General Condition Category |
| CDA | Finding | 2 | Review of Systems^*Gastrointestinal | Genitourinary Type Category |
| CDA | Finding | 2 | Neurologic examination | Hearing Loss Location Category |
| CDA | Finding | 2 | Symptom^*Sign | Heart Disease Symptom and Sign Category |
| CDA | Finding | 2 | Physical examination | Height of Nutritional Evaluation Measurement |
| CDA | Finding | 2 | Review of systems^*HEENT | HEENT Type Category |
| CDA | Finding | 2 | Neurologic Examination | Infant and Adolescent Intelligence Type Category |
| CDA | Finding | 2 | *Physical examination^Diagnosis | Infant and Adolescent Exercise Development Type Category |
| CDA | Finding | 2 | *Physical examination^Diagnosis | Infant and Adolescent Growth Development (Height,Weight) Type Category |
| CDA | Finding | 2 | Test Results | Image, Test Result and Other Data Specify |
| CDA | Finding | 2 | Review of Systems | Important Points of ROS Description |
| CDA | Finding | 2 | Test Results | Instrument of Pain Evaluation Specify |
| CDA | Finding | 2 | Test Results | Intensity of Pain Evaluation Specify |
| CDA | Finding | 2 | Test Results | Intervention of Pain Evaluation Specify |
| CDA | Finding | 2 | Test Results | Location of Pain Evaluation Specify |
| CDA | Finding | 2 | Physical examination | Mass Type Category |
| CDA | Finding | 2 | Physical examination | Mass Yes or No indicator |
| CDA | Finding | 2 | Test Results | Major Ingredient of Toxic Element Specify |
| CDA | Finding | 2 | Test Results | Modality of Pain Evaluation Specify |
| CDA | Finding | 2 | Review of Systems | Musculoskeletal Type Category |
| CDA | Finding | 2 | Review of Systems | Neuromuscular Status Specify |
| CDA | Finding | 2 | Physical examination | Other Physical examination comment Specify |
| CDA | Finding | 2 | Physical examination | Other Physical Examination Specify |
| CDA | Finding | 2 | Neurologic examination | Other Abnormal Specify |
| CDA | Finding | 2 | Neurologic examination | Other Neurological Examination Result Specify |
| CDA | Finding | 2 | Test Results | Outpatient Pretreatment Required test Specify |
| CDA | Finding | 2 | Symptom | Pain Evaluation Type Category |
| CDA | Finding | 2 | *Symptom^Admission | Pain Yes or No Indicator |
| CDA | Finding | 2 | *Symptom^Skin | Pruritus, Skin Rash, Yes or No Indicator |
| CDA | Finding | 2 | Neurologic examination | Papilledema Location Category |
| CDA | Finding | 2 | Physical examination | PE(Physical Examination) Results |
| CDA | Finding | 2 | Physical examination | Pediatric Head Circumference (cm) |
| CDA | Finding | 2 | Physical examination | Pediatric Physical Examination No Specific Finding Indicator |
| CDA | Finding | 2 | Physical examination | Pediatric Thoracic Circumference (cm) |
| CDA | Finding | 2 | Physical examination | Period and Type of Weight Change Specify |
| CDA | Finding | 2 | Test Results | Period of Pain Evaluation Specify |
| CDA | Finding | 2 | *Finding by Image ^Abdomen | Physical Examination of Abdomen Image |
| CDA | Finding | 2 | *Finding by Image^Chest | Physical Examination of Chest Image |
| CDA | Finding | 2 | *Reflex^Finding by Image | Deep Tendon Reflex Image |
| CDA | Finding | 2 | Physical examination | Physical Examination Non Specific |
| CDA | Finding | 2 | Physical examination | Physical Examination Abnormal Type Category |
| CDA | Finding | 2 | Physical examination | Physical Examination Mental Status Category |
| CDA | Finding | 2 | Physical examination | Physical Examination Type Category |
| CDA | Finding | 2 | Neurologic examination | Ptosis Location Category |
| CDA | Finding | 2 | Physical examination | Rectal Examination Other Specify |
| CDA | Finding | 2 | Physical examination | Rectal Examination Result |
| CDA | Finding | 2 | Physical examination | Rectal Examination Yes or No Indicator |
| CDA | Finding | 2 | Neurologic examination | Release of Contracture of Sternomastoid Muscle Type Category |
| CDA | Finding | 2 | Physical examination | Skin Status Examination Result |
| CDA | Finding | 2 | Physical examination | Skin Status Examination Specify |
| CDA | Finding | 2 | Neurologic examination | Soft Palate Elevation Direction Category |
| CDA | Finding | 2 | Diagnosis | R/O Diagnosis Name Specify |
| CDA | Finding | 2 | Diagnosis | R/O Diagnosis Name of Postoperative Specify |
| CDA | Finding | 2 | Diagnosis | R/O Diagnosis Name of Preoperative Specify |
| CDA | Finding | 2 | *Social History^Diagnosis | The Old and Infirm Bathing Type Category |
| CDA | Finding | 2 | *Social History^Diagnosis | The Old and Infirm Meal Type Category |
| CDA | Finding | 2 | *Social History^Diagnosis | The Old and Infirm Movement Type Category |
| CDA | Finding | 2 | *Social History^Diagnosis | The Old and Infirm Toilet Use Type Category |
| CDA | Finding | 2 | *Social History^Diagnosis | The Old and Infirm Urine and Feces Control Type Category |
| CDA | Finding | 2 | *Social History^Diagnosis | The Old and Infirm Dressing Type Category |
| CDA | Finding | 2 | Review of Systems^*Respiratory History | Respiratory Status Specify |
| CDA | Finding | 2 | Review of Systems^*Respiratory History | Respiratory Type Category |
| CDA | Finding | 2 | Review of systems | ROS(Review of Systems) Result Specify |
| CDA | Finding | 2 | Review of systems | ROS(Review of Systems) Condition Category |
| CDA | Finding | 2 | Review of systems | ROS(Review of Systems) General Condition Category |
| CDA | Finding | 2 | Review of systems | ROS(Review of Systems) General Condition Specify |
| CDA | Finding | 2 | Review of systems | ROS(Review of Systems) HEENT Specify |
| CDA | Finding | 2 | Review of systems | ROS(Review of Systems) Other Specify |
| CDA | Finding | 2 | Symptom | Symptom Onset Time |
| CDA | Finding | 2 | Symptom | Symptom Onset Time Unidentified |
| CDA | Finding | 2 | Physical examination | Suture Status Specify |
| CDA | Finding | 2 | Physical examination^Chest | Tactile Fremitus Occurrence Type Category |
| CDA | Finding | 2 | Neurologic examination | Tinnitus Location Category |
| CDA | Finding | 2 | Neurologic examination | Tongue Deviation Direction Category |
| CDA | Finding | 2 | Neurologic examination | Tongue Fasciculation, Plus or Minus Indicator |
| CDA | Finding | 2 | Test Results | Toxic Element Specify |
| CDA | Finding | 2 | Surgery^*Complication | Intraoperative Complication Yes or No indicator |
| CDA | Finding | 2 | Neurologic examination | Uvular Deviation Direction Category |
| CDA | Finding | 2 | Physical examination | Waist Circumference Measurement (cm) |
| CDA | Finding | 2 | *Physical examination^ Review of systems | Weight Change Gap Specify |
| CDA | Finding | 2 | Physical examination | Weight of Nutritional Evaluation Measurement |
| CDA | Finding | 3 | Abdomen | Direct Tenderness Location Category |
| CDA | Finding | 3 | Abdomen^Pelvis | Abdomen and Pelvis Status Specify |
| CDA | Finding | 3 | Abdomen | Abdomen Tenderness Plus or Minus Indicator |
| CDA | Finding | 3 | Abdomen | Abdomen Type Category |
| CDA | Finding | 3 | Abdomen | Abdomen P/Ex Flat Specify |
| CDA | Finding | 3 | Abdomen | Abdomen P/Ex Soft Specify |
| CDA | Finding | 3 | Abdomen | Ascites Type Category |
| CDA | Finding | 3 | HEENT | Anemic Conjunctiva, Plus Minus Indicator |
| CDA | Finding | 3 | Laboratory tests | Activated Partial Thromboplastin Time Measurement |
| CDA | Finding | 3 | Function test | Activity Evaluation Type Category |
| CDA | Finding | 3 | Laboratory tests | Alanine Transaminase Measurement |
| CDA | Finding | 3 | Laboratory tests | Albumin Measurement |
| CDA | Finding | 3 | Laboratory tests | Alcohol Measurement |
| CDA | Finding | 3 | Laboratory tests | Alkaline Phosphatase Measurement |
| CDA | Finding | 3 | Laboratory tests | Amylase Measurement |
| CDA | Finding | 3 | Laboratory tests | Arterial Blood Gas Analysis |
| CDA | Finding | 3 | Laboratory tests | Aspartate Transaminase Measurement |
| CDA | Finding | 3 | Back and extremity | Back and Extremity LOM Yes or No Indicator |
| CDA | Finding | 3 | Back and extremity | Back and Extremity Other Specify |
| CDA | Finding | 3 | Back and Extremity | Back and Extremity Result |
| CDA | Finding | 3 | Back and extremity | CVA(costovertebral angle) Tenderness Occurrence Category |
| CDA | Finding | 3 | Back and extremity | Pitting Edema Location Category |
| CDA | Finding | 3 | Back and extremity | Extremity Status Description |
| CDA | Finding | 3 | Back and extremity | Grossly Status of Extremity Category |
| CDA | Finding | 3 | Pathologic findings | Biopsy Name Specify |
| CDA | Finding | 3 | Pathologic findings | Biopsy Performed Date Specify |
| CDA | Finding | 3 | Pathologic findings | Biopsy Finding Specify |
| CDA | Finding | 3 | Lymph node | Clavicular, Supraclavicular Lymph Node Status Category |
| CDA | Finding | 3 | HEENT | HEENT(Head, Ear, Eye, Nose and Throat) Status, No Specific Finding Indicator |
| CDA | Finding | 3 | HEENT | Icteric Sclera, Plus Minus Indicator |
| CDA | Finding | 3 | Mouth | Lip and Tongue Condition Specify |
| CDA | Finding | 3 | Lymph node | Lymph Node Location Category |
| CDA | Finding | 3 | HEENT | Neck Vein Status Category |
| CDA | Finding | 3 | HEENT | Palatine Tonsillar Hypertrophy Location Category |
| CDA | Finding | 3 | HEENT | Paranasal Sinus Tenderness Pharyngeal Injection Plus Minus Indicator |
| CDA | Finding | 3 | HEENT | Thyroid Enlargement, Plus or Minus Indicator |
| CDA | Finding | 3 | HEENT | Tongue Status Category |
| CDA | Finding | 3 | HEENT | Venous Engorgement Location Category |
| CDA | Finding | 3 | Lymph node | Palpable Cervical L/N Category |
| CDA | Finding | 3 | Lymph node | Palpable Cervical L/N Size Measurement |
| CDA | Finding | 3 | *Finding by Image ^Abdomen | Physical Examination of Abdomen Image |
| CDA | Finding | 3 | *Finding by Image^Chest | Physical Examination of Chest Image |
| CDA | Finding | 3 | Abdomen | Physical Examination of Abdomen Other Specify |
| CDA | Finding | 3 | Laboratory tests | Urine Color Test |
| CDA | Finding | 3 | Laboratory tests | Urine Cytology Test |
| CDA | Finding | 3 | Cranial nerves | Visual Disturbance Location Category |
| CDA | Finding | 3 | Cranial nerves | Visual Field Defect Location Category |
| CDA | Finding | 3 | Vital signs | Blood Pressure of Vital Sign Measurement |
| CDA | Finding | 3 | Laboratory tests | Blood Urea Nitrogen Measurement |
| CDA | Finding | 3 | Vital signs | Body Temperature of Vital Sign Measurement |
| CDA | Finding | 3 | Abdomen | Bowel Sound Type Category |
| CDA | Finding | 3 | Chest | Breath Sound Status Category |
| CDA | Finding | 3 | Chest | Breathing Sound Type Category |
| CDA | Finding | 3 | Laboratory tests | Calcium Measurement |
| CDA | Finding | 3 | Chest | Chest Expansion Type Category |
| CDA | Finding | 3 | Chest | Chest Other Specify |
| CDA | Finding | 3 | Chest | Chest Status Description |
| CDA | Finding | 3 | Radiology | Chest X-ray Specify |
| CDA | Finding | 3 | *Chest^Lung | Chest/Lung P/Ex Other Specify |
| CDA | Finding | 3 | *Chest^Lung | Chest/Lung Retraction, Yes or No Indicator |
| CDA | Finding | 3 | *Chest^Lung | Chest/Lung Status Type Category |
| CDA | Finding | 3 | *Chest^Lung | Chest/Lung Symmetric Expansion, Yes or No Indicator |
| CDA | Finding | 3 | *Chest^Lung | Chest/Lung Breath Sounds(BS) Status Specify |
| CDA | Finding | 3 | *Chest^Lung | Chest/Lung Heart Sounds Status Specify |
| CDA | Finding | 3 | *Chest^Lung | Chest/Lung Rhonchi, Yes or No Indicator |
| CDA | Finding | 3 | *Chest^Lung | Chest/Lung Wheezing, Yes or No Indicator |
| CDA | Finding | 3 | Chest^Lung | Chest/Lung P/Ex Other Specify |
| CDA | Finding | 3 | Chest^Lung | Chest/Lung Retraction, Yes or No Indicator |
| CDA | Finding | 3 | Chest^Lung | Chest/Lung Status Type Category |
| CDA | Finding | 3 | Chest^Lung | Chest/Lung Symmetric Expansion, Yes or No Indicator |
| CDA | Finding | 3 | Chest^Lung | Chest/Lung Breath Sounds(BS) Status Specify |
| CDA | Finding | 3 | Chest^Lung | Chest/Lung Heart Sounds Status Specify |
| CDA | Finding | 3 | Chest^Lung | Chest/Lung Rhonchi, Yes or No Indicator |
| CDA | Finding | 3 | Chest^Lung | Chest/Lung Wheezing, Yes or No Indicator |
| CDA | Finding | 3 | Coordination and gait | Gait Type Category |
| CDA | Finding | 3 | Coordination and gait | Tandem Gait Status Type |
| CDA | Finding | 3 | Laboratory tests | Chloride Measurement |
| CDA | Finding | 3 | Laboratory tests | CK(Creatinine Kinase) Measurement |
| CDA | Finding | 3 | Mental Status | Cognitive Impairment Status Category |
| CDA | Finding | 3 | Laboratory tests | Complete Blood Measurement Cell Count |
| CDA | Finding | 3 | Reflex | Corneal Reflex Location Category |
| CDA | Finding | 3 | Cranial nerves | Cranial Nerve Examination Result, No Specific Finding Indicator |
| CDA | Finding | 3 | Laboratory tests | C-reactive Protein Measurement |
| CDA | Finding | 3 | Laboratory tests | Creatinine Kinase MB Measurement |
| CDA | Finding | 3 | Laboratory tests | Creatinine Measurement |
| CDA | Finding | 3 | Radiology | CT/SONO/MRI Examination Finding Specify |
| CDA | Finding | 3 | Radiology | CXR Examination Finding Specify |
| CDA | Finding | 3 | *Reflex^Finding by Image | Deep Tendon Reflex Image |
| CDA | Finding | 3 | Back and extremity | Deformities Status Specify |
| CDA | Finding | 3 | Vital signs | Diastolic Blood Pressure (mmHg) Measurement |
| CDA | Finding | 3 | Laboratory tests | Direct Bilirubin Measurement |
| CDA | Finding | 3 | Reflex | Direct Light Reflex Specify |
| CDA | Finding | 3 | Function test | Disability Evaluation Type Category |
| CDA | Finding | 3 | Function test | Dix Hallpike Test Specify |
| CDA | Finding | 3 | Reflex | Doll's Eye Reflex, Plus or Minus Indicator |
| CDA | Finding | 3 | Reflex | Gag Reflex, Plus or Minus Indicator |
| CDA | Finding | 3 | Laboratory tests | Electrolyte Measurement |
| CDA | Finding | 3 | Function test | Evaluation of Specific Patients Type Category |
| CDA | Finding | 3 | Sensory | Facial Sense Specify |
| CDA | Finding | 3 | Sensory | Facial Palsy Location Category |
| CDA | Finding | 3 | Function test | Finger to Finger Test Result Clumsiness Location Type |
| CDA | Finding | 3 | Function test | G.C.S. (Glasgow Coma Scale) Eye Opening Score |
| CDA | Finding | 3 | Function test | G.C.S. (Glasgow Coma Scale) Motor Respiratory Score |
| CDA | Finding | 3 | Function test | G.C.S. (Glasgow Coma Scale) Total Sore |
| CDA | Finding | 3 | Function test | G.C.S. (Glasgow Coma Scale) Verbal Respiratory Score |
| CDA | Finding | 3 | Laboratory tests | Glomerular Filtration Rate Measurement |
| CDA | Finding | 3 | Laboratory tests | HbsAg/Ab Examination |
| CDA | Finding | 3 | Chest | Heart Beat Status Category |
| CDA | Finding | 3 | Heart | Heart Beat Specify |
| CDA | Finding | 3 | Chest | Heart Rhythm and Murmur Status Category |
| CDA | Finding | 3 | Heart | Heart type Category |
| CDA | Finding | 3 | Function test | Heel to Shin Test Result Clumsiness Location Type |
| CDA | Finding | 3 | Reflex | Indirect Light Reflex Specify |
| CDA | Finding | 3 | Vital signs | Initial Pulse Rate Not Measured Reason Specify |
| CDA | Finding | 3 | Vital signs | Initial Pulse Rate Measurement |
| CDA | Finding | 3 | Laboratory tests | Kalium Measurement |
| CDA | Finding | 3 | Pathologic findings | Lab Finding |
| CDA | Finding | 3 | Laboratory tests | Lactate Dehydrogenase Measurement |
| CDA | Finding | 3 | Laboratory tests | Leukocyte Esterase Test |
| CDA | Finding | 3 | Motor | Lower Left Motor Status Grade Category |
| CDA | Finding | 3 | Motor | Lower Left Motor, Plus or Minus Indicator |
| CDA | Finding | 3 | Sensory | Lower Left Sensory Status Category |
| CDA | Finding | 3 | Sensory | Lower Left Sensory Type Category |
| CDA | Finding | 3 | Motor | Lower Right Motor Status Grade Category |
| CDA | Finding | 3 | Motor | Lower Right Motor, Plus or Minus Indicator |
| CDA | Finding | 3 | Sensory | Lower Right Sensory Status Category |
| CDA | Finding | 3 | Sensory | Lower Right Sensory Type Category |
| CDA | Finding | 3 | Function test | Lower/Left Motor Nerve Status Specify |
| CDA | Finding | 3 | Function test | Lower/Left Sensory Nerve Status Specify |
| CDA | Finding | 3 | Function test | Lower/Right Motor Nerve Status Specify |
| CDA | Finding | 3 | Function test | Lower/Right Sensory Nerve Status Specify |
| CDA | Finding | 3 | Laboratory tests | Magnesium Measurement |
| CDA | Finding | 3 | Mental Status | Memory Disturbance Specify |
| CDA | Finding | 3 | Function test | Meningeal Irritation Sign Type Category |
| CDA | Finding | 3 | Mental Status | Mental Status Specify |
| CDA | Finding | 3 | Mental Status | Mental Status Examination Result |
| CDA | Finding | 3 | Function test | Motor Other Specify |
| CDA | Finding | 3 | Motor | Motor Status, No Specific Finding Indicator |
| CDA | Finding | 3 | Function test | Motor Pathologic Reflex Specify |
| CDA | Finding | 3 | Laboratory tests | Myoglobin Examination Result |
| CDA | Finding | 3 | Laboratory tests | Natrium Measurement |
| CDA | Finding | 3 | Neck | Neck Physical Examination Other Specify |
| CDA | Finding | 3 | Neck | Neck Status Examination Result |
| CDA | Finding | 3 | Neck | Neck Status Examination Specify |
| CDA | Finding | 3 | Neck | Neck vein Status Specify |
| CDA | Finding | 3 | Laboratory tests | Nitrite Test Specify |
| CDA | Finding | 3 | Abdomen | Organomegaly Type Category |
| CDA | Finding | 3 | Mental Status | Orientation Status Type |
| CDA | Finding | 3 | Cranial nerves | Other Cranial Nerve Examination Result Specify |
| CDA | Finding | 3 | Motor | Other Motor Status Specify |
| CDA | Finding | 3 | Sensory | Other Sensory Status Specify |
| CDA | Finding | 3 | Endocrine History^*HEENT | Parathyroid Hormone Status Specify |
| CDA | Finding | 3 | Back and extremity | Paresthesia, Yes or No Indicator |
| CDA | Finding | 3 | Back and extremity | Peripheral Status Category |
| CDA | Finding | 3 | Neck | Pharyngeal Injection Specify |
| CDA | Finding | 3 | Laboratory tests | Phenyl Ketonuria Test |
| CDA | Finding | 3 | Laboratory tests | Prothrombin Time Measurement |
| CDA | Finding | 3 | Vital signs | Pulse of Vital Sign Measurement |
| CDA | Finding | 3 | Radiology | Radiologic Finding |
| CDA | Finding | 3 | Chest | Rale Occurrence |
| CDA | Finding | 3 | Chest | Rale Location Category |
| CDA | Finding | 3 | Function test | Rapid Alternating Movement Clumsiness Location Type |
| CDA | Finding | 3 | Abdomen | Rebound Tenderness Location Category |
| CDA | Finding | 3 | Reflex | Reflex Direct of Lt. Pupil Plus or Minus Indicator |
| CDA | Finding | 3 | Reflex | Reflex Direct of Rt. Pupil Plus or Minus Indicator |
| CDA | Finding | 3 | Reflex | Reflex Indirect of Lt. Pupil Plus or Minus Indicator |
| CDA | Finding | 3 | Reflex | Reflex Indirect of Rt. Pupil Plus or Minus Indicator |
| CDA | Finding | 3 | Reflex | Reflex Status, No Specific Finding Indicator |
| CDA | Finding | 3 | Vital signs | Respiratory of Vital Sign Measurement |
| CDA | Finding | 3 | Function test | Romberg Sign, Plus or Minus Indicator |
| CDA | Finding | 3 | Sensory | Sensory Status, No Specific Finding Indicator |
| CDA | Finding | 3 | Spine | Spine Status Specify |
| CDA | Finding | 3 | Vital signs | Systolic Blood Pressure (mmHg) Measurement |
| CDA | Finding | 3 | Laboratory tests | T. Cholesterol Measurement |
| CDA | Finding | 3 | Laboratory tests | Tone Type Category |
| CDA | Finding | 3 | Laboratory tests | Total Bilirubin Measurement |
| CDA | Finding | 3 | Laboratory tests | Total Protein Measurement |
| CDA | Finding | 3 | Laboratory tests | Troponin Examination |
| CDA | Finding | 3 | Motor | Upper Left Motor Status Grade Category |
| CDA | Finding | 3 | Motor | Upper Left Motor, Plus or Minus Indicator |
| CDA | Finding | 3 | Sensory | Upper Left Sensory Status Category |
| CDA | Finding | 3 | Sensory | Upper Left Sensory Type Category |
| CDA | Finding | 3 | Motor | Upper Right Motor Status Grade Category |
| CDA | Finding | 3 | Motor | Upper Right Motor, Plus or Minus Indicator |
| CDA | Finding | 3 | Sensory | Upper Right Sensory Status Category |
| CDA | Finding | 3 | Sensory | Upper Right Sensory Type Category |
| CDA | Finding | 3 | Motor | Upper/ Right Motor Nerve Status Specify |
| CDA | Finding | 3 | Motor | Upper/Left Motor Nerve Status Specify |
| CDA | Finding | 3 | Sensory | Upper/Left Sensory Nerve Status Specify |
| CDA | Finding | 3 | Sensory | Upper/Right Sensory Nerve Status Specify |
| CDA | Finding | 3 | Laboratory tests | Uric Acid Measurement |
| CDA | Finding | 3 | Laboratory tests | Urine Bilirubin Test |
| CDA | Finding | 3 | Laboratory tests | Urine Clarity Test |
| CDA | Finding | 3 | Laboratory tests | Urine Culture Test |
| CDA | Finding | 3 | Laboratory tests | Urine Occult Blood Test |
| CDA | Finding | 3 | Laboratory tests | Urine PH Measurement |
| CDA | Finding | 3 | Laboratory tests | Urine Protein Measurement |
| CDA | Finding | 3 | Laboratory tests | Urobilinogen Test |
| CDA | Finding | 3 | Laboratory tests | Venereal Disease Research Laboratory Test |
| CDA | Finding | 3 | Vital signs | Vital Sign Specify |
| CDA | Finding | 3 | Vital signs | Vital Sign Type Category |
| CDA | Finding | 4 | Heart function test | Cardiac Profile Test |
| CDA | Finding | 4 | Head | Head Status Examination |
| CDA | Finding | 4 | Head | Head Status Specify |
| CDA | Finding | 4 | Pulmonary function tests | Pulmonary Function Test Specify |
| CDA | Finding | 4 | Throat | Throat Status Examination Result |
| CDA | Finding | 4 | Throat | Throat Status Examination Specify |
| CDA | Finding | 4 | Throat | Throat Type Category |
| CDA | Finding | 4 | Eyes | Conjunctiva Status Category |
| CDA | Finding | 4 | Eyes | Conjunctiva Status Specify |
| CDA | Finding | 4 | Eyes | Eye Status Examination Result |
| CDA | Finding | 4 | Eyes | Eye Status Examination Specify |
| CDA | Finding | 4 | Eyes | Left Pupil Reflex Specify |
| CDA | Finding | 4 | Eyes | Pupil Size(R,L) Measurement |
| CDA | Finding | 4 | Eyes | Right Pupil Reflex Specify |
| CDA | Finding | 4 | Eyes | Sclera Status Category |
| CDA | Finding | 4 | Eyes | Sclera Status Specify |
| CDA | Finding | 5 | Electrocardiogram | Electrocardiogram Specify |
| CDA | Procedure | 1 | Procedure | Activated Charcoal Type Specify |
| CDA | Procedure | 1 | Procedure | Continuous Renal Replacement Therapy Setting Specify |
| CDA | Procedure | 1 | Procedure | Extracorporeal Treatment Type Category |
| CDA | Procedure | 1 | Procedure | Gastric Decontamination Type Category |
| CDA | Procedure | 1 | Procedure | Gastric Lavage Via Category |
| CDA | Procedure | 1 | Procedure | Gastric Lavage Volume |
| CDA | Procedure | 1 | Procedure | Hemoperfusion Performed Specify |
| CDA | Procedure | 1 | *Procedure^Surgery | Intraoperative Sended to Laboratory Culture Yes or No Indicator |
| CDA | Procedure | 1 | *Procedure^Surgery | Intraoperative Sended to laboratory Cytology Yes or No indicator |
| CDA | Procedure | 1 | *Procedure^Surgery | Intraoperative Sended to Laboratory Specimen Yes or No Indicator |
| CDA | Procedure | 1 | Procedure | Skin Incision Specify |
| CDA | Procedure | 1 | Procedure | Intraoperative Specific Facts Specify |
| CDA | Procedure | 1 | Procedure | Operation and Treatment Name |
| CDA | Procedure | 1 | Procedure | Operation Impression and Procedure Specify |
| CDA | Procedure | 2 | Surgery | Combine Op. Specify |
| CDA | Procedure | 2 | Anesthesia procedures | Anesthesia Type Category |
| CDA | Procedure | 2 | Anesthesia procedures | Anesthesia Type Specify |
| CDA | Procedure | 2 | Anesthesia procedures^ *Demographics | Anesthesia Doctor's Name Specify |
| CDA | Procedure | 2 | Discharge^*Diet | Dietetic Theraphy Specify |
| CDA | Procedure | 2 | Discharge^*Prescription procedure | Discharge Medicine Dose Period Specify |
| CDA | Procedure | 2 | Discharge^*Prescription procedure | Discharge Medicine Name Specify |
| CDA | Procedure | 2 | Discharge^*Prescription procedure | Discharge Medicine Usage Specify |
| CDA | Procedure | 2 | *Follow-up^Plan | F/U Plan Specify |
| CDA | Procedure | 2 | Dialysis procedure | Hemodialysis Performed Specify |
| CDA | Procedure | 2 | Surgery^*Complication | Intraoperative Complication Yes or No indicator |
| CDA | Procedure | 2 | Treatment^*Care Plan | Comment of care plan Specify |
| CDA | Procedure | 2 | Surgery | Intraoperative Total Bleeding |
| CDA | Procedure | 2 | Diet | Last Meal Specify |
| CDA | Procedure | 2 | Discharge^*Prescription procedure | Medication Dose Frequency |
| CDA | Procedure | 2 | Discharge^*Prescription procedure | Medication Quantity |
| CDA | Procedure | 2 | Discharge^*Prescription procedure | Medication Unit |
| CDA | Procedure | 2 | *Procedure^Surgery | Intraoperative Sended to Laboratory Culture Yes or No Indicator |
| CDA | Procedure | 2 | *Procedure^Surgery | Intraoperative Sended to laboratory Cytology Yes or No indicator |
| CDA | Procedure | 2 | *Procedure^Surgery | Intraoperative Sended to Laboratory Specimen Yes or No Indicator |
| CDA | Procedure | 2 | Surgery^*Demographics | Nursing Name Specify |
| CDA | Procedure | 2 | Surgery^*Demographics | Surgeon's Name Specify |
| CDA | Procedure | 2 | Surgery | Operation and Treatment Date |
| CDA | Procedure | 2 | Surgery | Operation Department Specify |
| CDA | Procedure | 2 | Surgery | Operation Method Specify |
| CDA | Procedure | 2 | Surgery | Operation Summary |
| CDA | Procedure | 2 | Surgery | Operation Time |
| CDA | Procedure | 2 | Surgery | Operation Type |
| CDA | Procedure | 2 | Surgery | Operation the Number of Times |
| CDA | Procedure | 2 | Surgery | Performed Date of Operation and Aids |
| CDA | Procedure | 2 | Plan | Plan Description |
| CDA | Procedure | 2 | Plan | Plan Yes or No Indicator |
| CDA | Procedure | 2 | Progress^Plan | Treatment Progress and Plan Specify |
| CDA | Procedure | 2 | Transfusion | Transfusion of Past History Yes or No Indicator |
| CDA | Procedure | 2 | Treatment | Treatment Finding Other Specify |
| CDA | Procedure | 2 | Treatment | Treatment Finding Type Category |
| CDA | Procedure | 2 | Treatment | Treatment Plan Type Category |
| CDA | Procedure | 2 | Treatment | Treatment Result Type Category |
| CDA | Procedure | 2 | Treatment | Treatment Result Category |
| CDA | Procedure | 2 | *Treatment^Discharge | Treatment Result Specify |
| CDA | Procedure | 2 | *Treatment^Outpatient Clinic | Outpatient Treatment Test Date Specify |
| CDA | Procedure | 2 | Treatment | Treatment Specify |
| CDA | Procedure | 3 | Radiotherapy | CT/SONO/MRI Examination Name Specify |
| CDA | Procedure | 3 | Radiotherapy | CT/SONO/MRI Performed Date Specify |
| CDA | Procedure | 3 | Radiotherapy | CXR Name Specify |
| CDA | Procedure | 3 | Radiotherapy | CXR Perform Date Specify |
| CDA | Procedure | 3 | Current medication^*Follow-Up | Medicine and Treatment Follow Up Specify |
| CDA | Procedure | 3 | Current medication | Medicine Treatment Related Matters Specify |
| CDA | Procedure | 3 | Current medication | Present Taking Medicine and Taking Medical Treatment Type Category |
| CDA | Procedure | 3 | Current medication | Present Taking Drug Name Specify |
| CDA | Procedure | 3 | Care Plan | Goal of Care Plan Specify |
| CDA | Procedure | 3 | Care Plan | Goal Date of Care Plan Specify |
| CDA | Procedure | 3 | Care Plan | Problem of Care Plan Specify |
| CDA | Procedure | 3 | *Follow-up^Care Plan | F/U Care Plan Associated Comment Specify |
| CDA | Procedure | 3 | Education Plan | Education Plan Date Specify |
| CDA | Procedure | 3 | Education Plan | Content of Education Plan Specify |
| CCR+ | Description | 2 | Advance Directives | AdvanceDirectives |
| CCR+ | Description | 2 | Advance Directives | AdvanceDirective DateTime |
| CCR+ | Description | 2 | Advance Directives | AdvanceDirective DateTime Type |
| CCR+ | Description | 2 | Advance Directives | AdvanceDirective Specify |
| CCR+ | Description | 2 | Advance Directives | AdvanceDirectivescription Text |
| CCR+ | Description | 2 | Advance Directives | AdvanceDirective Status |
| CCR+ | Description | 2 | Advance Directives | AdvanceDirective Type |
| CCR+ | Description | 2 | Advance Directives | Status of patient |
| CCR+ | Description | 2 | Alerts | Alerts |
| CCR+ | Description | 2 | Alerts | Alert Specify |
| CCR+ | Description | 2 | Alerts | Alert DateTime |
| CCR+ | Description | 2 | Alerts | Alert DateTime Type |
| CCR+ | Description | 2 | Alerts | Alert Reaction |
| CCR+ | Description | 2 | Alerts | Alert Reaction Description Text |
| CCR+ | Description | 2 | Alerts | Alert Reaction Intervention |
| CCR+ | Description | 2 | Alerts | Alert Reaction Severity |
| CCR+ | Description | 2 | Alerts | Alert Status |
| CCR+ | Description | 2 | Alerts | Alert Type |
| CCR+ | Description | 2 | Encounter | Consent Specify |
| CCR+ | Description | 2 | Encounter | Encounters |
| CCR+ | Description | 2 | Encounter | Encounter DateTime |
| CCR+ | Description | 2 | Encounter | Encounter DateTime Type |
| CCR+ | Description | 2 | Encounter | Encounter Specify |
| CCR+ | Description | 2 | Encounter | Encounter Status |
| CCR+ | Description | 2 | Encounter | Encounter Department |
| CCR+ | Description | 2 | Encounter | Encounter Type |
| CCR+ | Description | 2 | Encounter | Location Specify |
| CCR+ | Description | 2 | History of Immunization | Immunizations |
| CCR+ | Description | 2 | History of Immunization | Immunization DateTime |
| CCR+ | Description | 2 | History of Immunization | Immunization DateTime Type |
| CCR+ | Description | 2 | History of Immunization | Immunization Specify |
| CCR+ | Description | 2 | History of Immunization | Immunization Direction |
| CCR+ | Description | 2 | History of Immunization | Immunization Product |
| CCR+ | Description | 2 | History of Immunization | Immunization Type |
| CCR+ | Description | 3 | Payor | Payers |
| CCR+ | Description | 3 | Payor | Payer DateTime |
| CCR+ | Description | 3 | Payor | Payer DateTime Type |
| CCR+ | Description | 3 | Payor | Payer Specify |
| CCR+ | Description | 3 | Payor | Payer Type |
| CCR+ | Description | 3 | Payor | Payment Provider |
| CCR+ | Description | 3 | Payor | Subscriber Specify |
| CCR+ | Description | 3 | Family History | FamilyHistory |
| CCR+ | Description | 3 | Family History | FamilyMember |
| CCR+ | Description | 3 | Family History | FamilyMember HealthStatus DateTime |
| CCR+ | Description | 3 | Family History | FamilyMember HealthStatus DateTime Type |
| CCR+ | Description | 3 | Family History | FamilyProblemHistory Specify |
| CCR+ | Description | 3 | Family History | FamilyProblemHistory Problem |
| CCR+ | Description | 3 | Family History | FamilyProblemHistory Status |
| CCR+ | Description | 3 | Family History | FamilyProblemHistory Type |
| CCR+ | Description | 3 | Family History | HealthStatus DateTime |
| CCR+ | Description | 3 | Family History | HealthStatus DateTime Type |
| CCR+ | Description | 3 | Family History | HealthStatus Specify |
| CCR+ | Description | 3 | Medication History | Direction Dose |
| CCR+ | Description | 3 | Medication History | Direction Dose Unit |
| CCR+ | Description | 3 | Medication History | Medication Specify |
| CCR+ | Description | 3 | Medication History | Medication Direction |
| CCR+ | Description | 3 | Medication History | Medication PatientInstruction |
| CCR+ | Description | 3 | Medication History | Medication Product |
| CCR+ | Description | 3 | Medication History | Medication Quantity |
| CCR+ | Description | 3 | Medication History | Medication Refill |
| CCR+ | Description | 3 | Medication History | Medication Status |
| CCR+ | Description | 3 | Medication History | Direction Frequency |
| CCR+ | Description | 3 | Medication History | Direction Indication |
| CCR+ | Description | 3 | Medication History | Direction Indication LinkID |
| CCR+ | Description | 3 | Medication History | Direction Route |
| CCR+ | Description | 3 | Medication History | FulfillmentHistory |
| CCR+ | Description | 3 | Medication History | FulfillmentHistory Quantity |
| CCR+ | Description | 3 | Medication History | Medication Instruction |
| CCR+ | Description | 3 | Medication History | Medications |
| CCR+ | Description | 3 | Medication History | Medication DateTime |
| CCR+ | Description | 3 | Medication History | Medication DateTime Type |
| CCR+ | Description | 3 | Medication History | Medication Type |
| CCR+ | Description | 3 | Medication History | ProductName Specify |
| CCR+ | Description | 3 | Medication History | Product Form |
| CCR+ | Description | 3 | Medication History | Product Strength |
| CCR+ | Description | 3 | Social History | SocialHistoryElement DateTime |
| CCR+ | Description | 3 | Social History | SocialHistoryElement DateTime Type |
| CCR+ | Description | 3 | Social History | SocialHistoryElement |
| CCR+ | Description | 3 | Social History | SocialHistoryElement Status |
| CCR+ | Description | 3 | Social History | SocialHistory Specify |
| CCR+ | Description | 3 | Social History | SocialHistory Type |
| CCR+ | Finding | 2 | Problem | Active Inactive Indicator |
| CCR+ | Finding | 2 | Problem | CauseOfDeath |
| CCR+ | Finding | 2 | Problem | HealthStatus in Social History |
| CCR+ | Finding | 2 | Problem | Problems |
| CCR+ | Finding | 2 | Problem | Problem DateTime |
| CCR+ | Finding | 2 | Problem | Problem DateTime Type |
| CCR+ | Finding | 2 | Problem | Problem Specify |
| CCR+ | Finding | 2 | Problem | Problem Status |
| CCR+ | Finding | 2 | Problem | Problem Severity |
| CCR+ | Finding | 2 | Problem | Problem Type |
| CCR+ | Finding | 2 | Problem | Status of treatment |
| CCR+ | Finding | 2 | Test Results | Results |
| CCR+ | Finding | 2 | Test Results | Results Units |
| CCR+ | Finding | 2 | Test Results | Result Type |
| CCR+ | Finding | 2 | Test Results | Result DateTime |
| CCR+ | Finding | 2 | Test Results | Result DateTime Type |
| CCR+ | Finding | 2 | Test Results | Result Test |
| CCR+ | Finding | 2 | Test Results | Test DateTime |
| CCR+ | Finding | 2 | Test Results | Test DateTime Type |
| CCR+ | Finding | 2 | Test Results | Test Flag |
| CCR+ | Finding | 2 | Test Results | Test Status |
| CCR+ | Finding | 3 | Function Test Results | FunctionalStatus |
| CCR+ | Finding | 3 | Function Test Results | Function Title Specify |
| CCR+ | Finding | 3 | Function Test Results | Function Problem |
| CCR+ | Finding | 3 | Function Test Results | Function Result |
| CCR+ | Finding | 3 | Function Test Results | Function Status |
| CCR+ | Finding | 3 | Function Test Results | Function Test |
| CCR+ | Finding | 3 | Function Test Results | Function Type |
| CCR+ | Finding | 3 | Function Test Results | Test ActorID |
| CCR+ | Finding | 3 | Function Test Results | Test ActorRole |
| CCR+ | Finding | 3 | Function Test Results | Test Source |
| CCR+ | Finding | 3 | Function Test Results | NormalResult Range Cagetory |
| CCR+ | Finding | 3 | Function Test Results | Result Type |
| CCR+ | Finding | 3 | Function Test Results | TestResult |
| CCR+ | Finding | 3 | Vital Signs | VitalSigns |
| CCR+ | Finding | 3 | Vital Signs | VitalSigns Result |
| CCR+ | Finding | 3 | Vital Signs | VitalSign Specify |
| CCR+ | Finding | 3 | Vital Signs | VitalSign Type |
| CCR+ | Finding | 3 | Vital Signs | VitalSign Unit |
| CCR+ | Procedure | 1 | Procedure | Practitioners |
| CCR+ | Procedure | 1 | Procedure | Procedure DateTime |
| CCR+ | Procedure | 1 | Procedure | Practitioner ActorID |
| CCR+ | Procedure | 1 | Procedure | Practitioner ActorRole |
| CCR+ | Procedure | 1 | Procedure | Procedures |
| CCR+ | Procedure | 1 | Procedure | Procedure DateTime Type |
| CCR+ | Procedure | 1 | Procedure | Procedure Specify |
| CCR+ | Procedure | 1 | Procedure | Procedure Status |
